# Can behavioral science advance breastfeeding-friendly primary care? Key findings from an evaluation in Kosovo

**DOI:** 10.1371/journal.pgph.0005276

**Published:** 2025-10-31

**Authors:** Melinda McKay, Hana Bucinca, Wolf Peter-Schmidt, Tanya Marchant, Robert Aunger

**Affiliations:** 1 Department of Disease Control, London School of Hygiene & Tropical Medicine, London, United Kingdom; 2 Action for Mothers and Children, Pristina, Kosovo; The Ohio State University, UNITED STATES OF AMERICA

## Abstract

Breastfeeding is a cost-effective intervention vital to child health, yet rates remain suboptimal, especially in fragile settings. Primary care providers are critical to improving breastfeeding outcomes but face multilevel barriers. While hospital-based interventions have shown success, adaptations to primary care are limited, and understanding of the mechanisms driving provider behavior change remains insufficient. In Kosovo, where exclusive breastfeeding is declining alongside increasing infant mortality, we piloted a behavioral science intervention to improve primary care provider practices. A before-and-after design (baseline June–July 2019; endline June–July 2021) using mixed-methods assessed changes in provider behavior, maternal experience, and the institutional environment across five municipalities. Validation compared maternal reports with clinical observations. The intervention targeted provider behavior with emotionally engaging activities, job aids and institutional strategies, and was co-designed with system actors. Data sources included direct observation of consultations, exit interviews with mothers, provider interviews and facility assessments. Across 609 consultations with matched exit interviews, we recorded a 14.9 percentage point increase in the mean frequency of breastfeeding-friendly *clinical* behaviors by providers (95% CI: 6.4-23.4) and a 13.3 percentage point increase in *interpersonal* behaviors (95% CI: 5.2-21.3) after versus before intervention. Gains were strongest for providers explaining ability to breastfeed (+39.0 points), inviting mother to ask questions (+21.8 points) and explaining follow-up required (+18.7 points). Gaps between observer and maternal reports narrowed across most indicators, indicating stronger alignment between care delivered and perceived. Mothers reported greater confidence and better care experiences. Providers’ knowledge and attitudes improved and shifts in leadership engagement and facility norms were seen, though broader system integration was limited. A behaviorally informed, co-designed intervention can measurably improve breastfeeding counseling behaviors and maternal experiences in Kosovo’s primary care settings. Findings underscore the value of targeting deeper behavioral drivers, not just knowledge gaps, within fragile health systems.

## Introduction

Breastfeeding is one of the most cost-effective public health interventions, with profound impacts on child survival and long-term health outcomes. Yet globally, rates remain below World Health Organization (WHO) and United Nations Children’s Fund (UNICEF) recommendations, leading to preventable health and economic burdens [1–3]. This gap is especially critical in fragile and conflict-affected states, where maternal and neonatal conditions contribute to the greatest number of Disability Adjusted Life Years [4]. Scaling up breastfeeding practices in these settings is a public health priority.

Primary healthcare providers (doctors and nurses) are central to this effort. They have frequent interactions with mothers during antenatal, postnatal, and infant care visits [5], and improving their practices is associated with better breastfeeding outcomes [6]. Strengthening primary care provider practice is especially critical in low- and middle-income countries, which bear a 50-fold higher burden of morbidity and mortality from suboptimal breastfeeding compared to high-income countries [7]. WHO recommends at least six breastfeeding counseling contacts, most delivered in primary or community settings [8], highlighting the critical role of the primary care system.

Although hospital-based interventions like the Baby Friendly Hospital Initiative (BFHI) have shown that provider behavior change can improve breastfeeding rates [9–13], they primarily focus on the birth period and do not address the steep decline in breastfeeding continuation [14,15]. While there are efforts to adapt the BFHI to primary/community care settings, there is limited evidence regarding the quality of adaptations and their impact on provider behavior, maternal experiences, and institutional norms [15,16].

A synthesis of the literature exploring facilitators and barriers to adopting breastfeeding-friendly practice by providers in healthcare settings identifies factors at multiple levels [10, 16–41]. At the individual level, training, motivation, positive attitudes, and tapping into motivational drivers (e.g., perceived efficacy, emotional connection to clients) enable adoption, while barriers include knowledge gaps, resistance to change, time constraints, and norms (both organizational and social, e.g., a preference for formula feeding). At the institutional and structural level, political will and leadership, funding, interprofessional and community collaboration, and positive organizational norms support implementation, but resource gaps, weak leadership, lack of integration across different system levels and with broader health initiatives, along with formula industry influence pose challenges.

Insights from a formative research study for the primary care breastfeeding intervention in Kosovo were consistent with the literature. Increasing provider knowledge alone typically does not translate into practice change [41], however behavior change strategies often prioritize easily measurable outputs, such as training [41,42]. Applying behavioral science to provider behavior change is essential for uncovering underlying drivers, social influences, and systemic constraints [43–47], with the aim of fostering more effective and sustainable change strategies.

A novel intervention designed to support primary care providers in adopting breastfeeding-friendly practices was piloted in Kosovo. This paper presents the findings of the before-and-after, mixed-methods evaluation of the intervention to assess changes in clinical and interpersonal practices of providers during routine consultations (primary outcome of interest). To understand why, or why not, the intervention changed provider behaviors we also describe secondary outcomes of interest, including changes in maternal experience of care, provider knowledge and attitudes, and the institutional environment. By applying a behavioral science lens, the study sought to measure changes in provider practices and explore the underlying drivers of that change.

## Materials and methods

### Study setting

Kosovo has a neonatal mortality rate four times higher than the European average [48,49] and low and declining breastfeeding rates, with only 29% of infants under six months being exclusively breastfed, compared to the global average of 44% [48,50]. Kosovo provides statutory maternity leave and some provision for breastfeeding breaks on return to work, though both are inconsistently applied. Facility-level supports such as pumps or designated spaces are very limited. These broader entitlements form part of the breastfeeding context but were beyond the scope of this evaluation, which focused on provider counselling practices in primary care. Despite the high frequency of infant care provided within its public primary healthcare system, there is limited research on the frequency and quality of breastfeeding counseling, making it challenging to determine where and how to enhance support. At the primary care level, 29 out of Kosovo’s 38 municipalities have a “main” Family Medicine Center (FMC), which delivers diagnostic and curative care, including antenatal and postnatal services as well as immunizations. Smaller FMCs serve catchment populations of approximately 10,000 people, and in certain municipalities, they are the focal point for infant immunizations.

### Description of intervention

**Fig 1:**
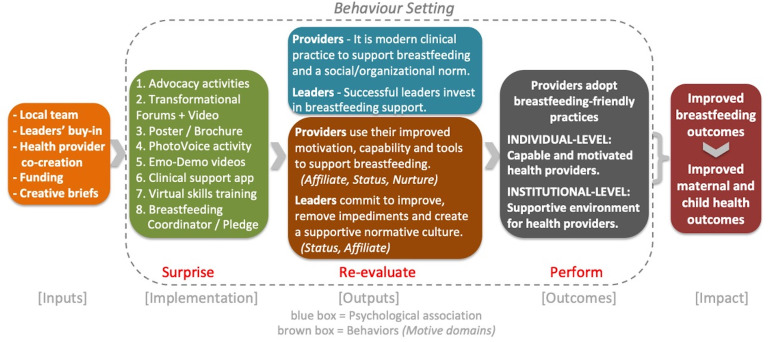
Theory of Change.

This study was conducted in one of Europe’s youngest and poorest countries, a fragile state with declining breastfeeding rates and rising infant mortality [48,49,51]. It was implemented in collaboration with Kosovar non-governmental organization Action for Mothers and Children (AMC), and combined a behavioral science framework, Behaviour Centred Design (BCD) [52], with human-centered design (HCD) to ensure provider-driven solutions and adaptive implementation. The theory of change (Fig 1) hypothesized that a behavioral-science grounded intervention, with multiple reinforcing activities co-designed with system actors, would establish breastfeeding-friendly practice as individual and institutional norms. This taps into specific behavioral drivers for providers and leaders, motivating them to apply their improved skills, tools and commitment to improvement, fostering a supportive culture and resulting in adoption of breastfeeding-friendly practices that ultimately improves breastfeeding outcomes (see Fig 1). To our knowledge, this is the first study to evaluate quantifiable behavioral outcomes alongside the mechanisms of change for breastfeeding-friendly practices in this type of setting.

Illustrates hypothesized mechanisms of change, linking intervention activities to motivational drivers (status, nurture, affiliation) and environmental cues. Figure adapted from [52]; available under CC-BY 4.0.

The intervention aimed to address individual and institutional barriers to change by disrupting existing behavioral settings and activating provider’s motivated and reactive mechanisms to facilitate changes in practice. Drawing on insights from formative research, the intervention targeted motives that matter to providers; *nurture*, *status*, and *affiliation*. Key intervention components included advocacy, inspirational away days (facilitating *affiliation* and shared norms) and motive-triggering virtual training for providers, clinical support tools, and emotionally engaging actions such as PhotoVoice and Emotional Demonstration (Emo-Demo) videos (activating *nurture* by illustrating empathetic care, and *status* by including influential clinicians modeling ideal behaviors). These elements were intentionally sequenced to shift providers’ perception of breastfeeding counseling from an undervalued task to a valued part of their professional role and identity. BCD provided the structure to align environmental cues, social norms, and individual motives into a reinforcing behavioral system. Table 1 summarizes the intervention activities and key considerations for their implementation; a paper that documents the intervention’s design and implementation has been submitted for publication. Each component of the final intervention was explicitly mapped to the barriers and facilitators identified during formative research and refined through HCD methods; see S1 File for the Logic Model. This mapping

**Table 1:** Matrix of intervention activities.

	1. Advocacy Activities				2. Forum + Video + Promotion			3. Poster/ Brochure					4. PhotoVoice Activity			
**Description**	A series of advocacy meetings and follow-up with leaders that makes the case for supporting breastfeeding and demonstrates an achievable pathway. Reinforced with a media strategy to motivate leaders, including a series of interviews with FMC Directors about the breastfeeding support offered at the FMC.				2x one-day inspiration event for 126 doctors and nurses from the sample FMCs, engineered to create “aha” moments via interactive activities led by a dynamic facilitator. Photos/videos were converted into a motivational video and distributed via closed Viber groups and AMC social media. A promotion strategy targeted providers and MOH via a range of approaches.			Creative infographic poster and brochure in Albanian, Serbian and English that includes the latest facts about breastfeeding and how/where to get support. It uses bold graphics to trigger surprise. The posters are for the walls of FMC waiting areas and the brochures for providers to give clients and to be available in FMC waiting areas.					Facilitated by the facility Breastfeeding Coordinators, staff were asked to capture in photo-form ‘what it means to support mothers and infants in your daily work, especially in relation to breastfeeding promotion.’ This activity generated dozens of photos that were used in the Emo-Demo videos, clinical app and skills training activities.			
**Goals**	Create a psychological shift that increases the value of breastfeeding in leaders’ mind and inspires them to support institutional change.				Create a psychological shift in providers that increases the value of breastfeeding in their mind, clarifies their role and inspires them to design and participate in a change pathway.			Surprise, engage and inform clients and staff. Remind providers and FMC Directors that breastfeeding support is a social norm at this FMC.					Spark provider thinking and bring them together around the issue. Add originality and relatability to videos/app/training and have providers model these behaviors.			
**Target Audience**	Nurses		Doctors	Leaders X	Nurses X	Doctors X	Leaders	Nurses X	Doctors X		Leaders X		Nurses X	Doctors X		Leaders
**Theory of Change role**	Surprise		Re-evaluate X	Perform X	Surprise X	Re-evaluate X	Perform X	Surprise X	Re-evaluate X		Perform		Surprise	Re-evaluate X		Perform X
	**5. Emo-Demo Videos**				**6. Clinical Support App**			**7. Skills Training**					**8. Breastfeeding Coordinator/ Pledge**			
**Description**	Three videos developed with providers to demonstrate how and why it is important to promote and support breastfeeding at every antenatal, infant care or vaccination consultation. 5,200 + views on AMC Facebook page, used in skills training, screened in some FMCs.				A modern, evidence-based job aid in Albanian on iOS/Android platforms for clinical staff in Kosovo. Endorsed by MOH, with data support from UNICEF and WHO. Includes a triage tool; medications guide; COVID-19 Q&A; videos for parents; feeding advice by age and other resources.			Four half-day Zoom-based training sessions in Albanian to equip 200 providers with the knowledge, tools, and motivation to support their FMC to be breastfeeding friendly via emotionally-resonant content and peer reflection. Certified by Doctors & Nurses Chambers of Kosovo.					Nomination of breastfeeding focal points at each facility by fellow staff + creation of a facility-level pledge for breastfeeding support adapted from the BFHI, developed with an national working group of key system actors, including facility leaders and breastfeeding coordinators.			
**Goals**	Spark change by connecting to what the formative research said motivates staff (*status*, *affiliation*, and *nurture*).				Make providing accurate breastfeeding advice easy, relevant and rewarding, thereby routine.			Increase confidence, capacity and interest to support breastfeeding. Reinforce professional collaboration.					Create the necessary institutional elements to support a breastfeeding-friendly culture at the facility.			
**Target Audience**	Nurses X	Doctors X		Leaders	Nurses X	Doctors X	Leaders	Nurses X		Doctors X		Leaders	Nurses X		Doctors X	Leaders X
**Theory of Change role**	Surprise X	Re-evaluate X		Perform	Surprise	Re-evaluate	Perform X	Surprise		Re-evaluate X		Perform X	Surprise		Re-evaluate X	Perform X

Description of each intervention component, target audience, goals, and role in the theory of change illustrates how barriers evolved through the co-design process into feasible strategies.

### Study design

**Fig 2:**
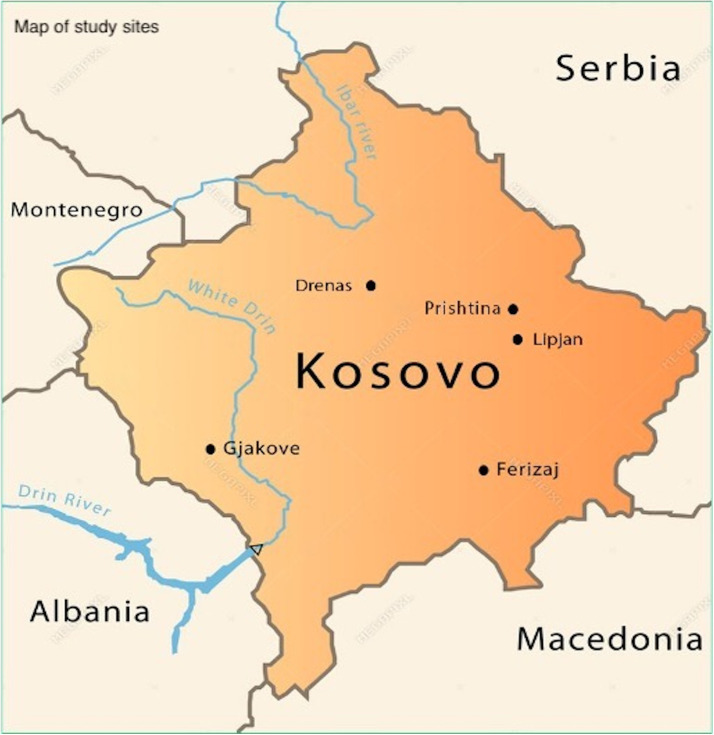
Map of study sites.

A before-and-after, mixed-methods design was implemented in seven FMCs (five “main” FMCs and two smaller FMCs) across five municipalities nationwide (see Fig 2). All selected facilities were urban-based and provided a similar range of services. Facilities were selected for geographic diversity and comparability of setting, services, and patient volumes in consultation with the study’s Advisory Committee. This committee included key actors in Kosovo’s health system, with representatives from the Ministry of Health, National Institute of Public Health Kosovo, BFHI national committee, WHO, UNICEF, and a primary care clinical trainer, along with a national design/marketing expert. Facilities were recruited through advocacy directed at facility directors that emphasized support from Advisory Committee members. Breastfeeding-friendly practices documented by trained observers during routine consultations with infants aged 0–12 months and their mothers at the FMC were assessed against maternal reports collected by exit interview immediately after the consultation.

Map of study sites across five municipalities in Kosovo. Redrawn specifically for this study by the authors’ designer. Republished from Action for Mothers and Children (AMC) under a CC BY license, with permission from AMC, original copyright 2019.

Routine consultations included postnatal checkups, acute care visits, routine care appointments, and immunization services for infants aged 12 months or younger. All providers attending to these consultations and on shift during the time of data collection were eligible for inclusion, along with mothers aged 18 years and older who attended these consultations with participating providers. Results compare the endline data (collected June-July 2021) against the baseline data (collected June-July 2019). The previously described intervention was implemented between December 2019 and April 2021, but was paused for ten months between March and December 2020 due to COVID-19. Therefore, active implementation of the intervention occurred over a non-consecutive period of seven months.

This study was reported in accordance with the STROBE (Strengthening the Reporting of Observational Studies in Epidemiology) checklist [53] to ensure comprehensive and transparent reporting of observational study elements.

### Data collection methods

Data collection comprised: i) observations of routine consultations for infants aged 0–12 months, ii) exit interviews with mothers immediately after the consultation, iii) one-on-one interviews with providers, and iv) general observations. Baseline data was collected in 2019 and endline data in 2021, each over the course of 4–5 days per site by 2–3 data collectors. Data collectors were independent of the study sites to minimize bias. To ensure participants’ comfort, all data collectors were female, fluent in Albanian and English, and had extensive training in research ethics, data collection methods, and the use of study tools. Their training included classroom instruction, role-playing, and facility-based testing. The same rigorous training was conducted before baseline and endline data collection, and some data collectors remained the same across both periods to maintain consistency.

Survey instruments were tested at a facility outside the study area before baseline data collection to confirm accurate translation from Albanian to English, evaluate interobserver agreement and confirm how mothers and providers interpreted questions, in accordance with WHO recommendations [54]. To minimize subjectivity in assessing interpersonal skills, clear definitions for each indicator were provided during the training of data collectors (see S2 File). Data was collected using paper forms and then entered into tablets by the final day at each site using ODKCollect [55]. Results were then uploaded directly to the London School of Hygiene & Tropical Medicine (LSHTM) server in encrypted format.

### Observations and exit interviews

During routine consultations, trained observers used a structured checklist (see S3 File) to evaluate the quality of breastfeeding counseling provided. Immediately after the consultation, exit interviews with mothers were conducted in a private area within the facility to minimize courtesy bias and recall issues. The exit interview tool included the same set of indicators as the observation checklist, adapted into question form for mothers (see S4 File). Observations were conducted by clinicians with research and breastfeeding counseling expertise, while exit interviews were carried out by medical students with prior research experience.

The eligibility criteria for observations were providers who attended to the consultation type of interest and working the day of data collection. Providers were recruited through a meeting called by the facility director at the start of day 1 at baseline and endline, where the lead data collector described the study (including its support from the MOH) and the facility director provided their endorsement. To reduce selection bias, a systematic observation approach was followed; each day consenting providers were randomly selected and followed for a set duration (typically 90–120 minutes), with all consultations that also received prior consent by mother within that timeframe assessed. This method was followed at both baseline and endline.

For exit interviews, the eligibility criteria were mothers, aged 18 years and older, who attended an observed consultation with a participating provider. Mothers were recruited by the lead data collector when the mother was called into the consultation room (and prior to starting the consultation). The source was all consultations observed, and consent to participate was verbally re-checked prior to starting the exit interview.

### Provider interviews

The same staff members who were observed during counseling also participated in one-on-one, face-to-face interviews conducted in a private setting using a structured form (see S5 File) by the same clinician that conducted the observations. Interviews were held after the observations/exit interviews to reduce bias. These methods were followed at both baseline and endline. The baseline survey was structured to take ±25 minutes, while the endline survey duration was planned for ±35 minutes due to the addition of process indicator questions. The eligibility criteria were providers whose consultations were observed during the data collection period. Invitation to interview was extended to all providers observed after the period of observation.

### General observations

On the first day of baseline data collection at each FMC, a guided tour was conducted by the head nurse to document the physical setting and confirm how care for mothers and infants was organized. The senior data collector asked structured questions, while the student data collector recorded observations in a field notes journal. At both baseline and endline, particular attention was given to facility layout, patient flow, available equipment, visual aids (such as posters or educational materials), and provider-client interactions. To supplement these observations, photographs were taken to document the physical space and general activity within the FMC, ensuring compliance with The General Data Protection Regulation (EU) 2016/679 [56]. These photographs were submitted to the study supervisor before the conclusion of data collection at each FMC. Throughout the baseline and endline data collection period, the team conducted non-intrusive observations of general facility activity, noting how providers interacted with each other and with clients outside of private consultations. Observations were recorded in the team’s field notes journals to capture recurring patterns, e.g., routines, and contextual factors influencing provider behavior. These were documented in the field notes journal to provide additional qualitative insights into the primary care environment.

### Data analysis and management

Indicators to measure the intervention effect were identified through a review of published and grey literature. WHO and UNICEF training materials were primarily used, and relevant survey instruments such as the Demographic and Health Survey [57] and the Multiple Indicator Cluster Survey [58] were referenced. The selection process was further refined and prioritized in collaboration with local experts. Two groups of primary outcome indicators and three groups of secondary outcome indicators were selected (see Table 2).

**Table 2:** Description of outcome measures. Definitions of primary and secondary outcome indicators used in the evaluation.

Indicator	Definition
**Primary Outcome Indicators**	
***CLINICAL BEHAVIORS:*** Mean frequency rate of breastfeeding- friendly clinical counseling practices by study providers during routine consultations after versus before intervention.	Combined mean frequency rate of the following eight practices during routine consultations in study facilities with infants aged 0–12 months, as reported by mothers (equal weighting): 1. Provider explained the benefits of breastfeeding. 2. Providers explained a woman’s ability to breastfeed. 3. Providers asked mother if she had breastfeeding questions. 4. Provider explained follow-up visits required. 5. Provider inquired about mothers’ support structure. 6. Provider gave take-home material about breastfeeding. 7. Provider explained breastfeeding support resources available. 8. Provider did not promote breastmilk substitutes (formula). 9. Provider observed mother breastfeeding.
***INTERPERSONAL BEHAVIORS:*** Mean frequency rate of breastfeeding- friendly interpersonal counseling practices by study providers during routine consultations after versus before intervention.	Combined mean frequency rate of the following three practices during routine consultations in study facilities with infants aged 0–12 months, as reported by mothers (equal weighting): 1. Provider really listened to/understood concerns of mother. 2. Provider made mother feel comfortable to express her opinions, feelings and concerns. 3. Provider explained things well and gave practical help in a way mother could understand.
**Secondary Outcome Indicators**	
***Maternal experience of care:*** Mean frequency rates of maternal experience of care and feelings about breastfeeding after versus before intervention.	Mean frequency rates of experience of care measured by: 1. Validation agreement rates of clinical and interpersonal counseling practices after versus before intervention. 2. Mean frequency of ‘good’/‘very good’ for maternal overall experience at the facility after versus before intervention. Mean frequency rate of ‘a lot’ or ‘a great deal’ for maternal assessment of breastfeeding’s value, intent and self-efficacy after versus before intervention based on four indicators.
***Providers’ knowledge and attitudes:*** Mean knowledge and attitude rates after versus before intervention.	Mean frequency rates of positive knowledge and attitudes as reported by interviewers or providers, based on the following: 1. Provider breastfeeding knowledge. 2. Provider attitudes towards breastfeeding. 3. Provider self-efficacy scores. 4. Provider opinion of influencers on mother’s breastfeeding.
***Institutional environment:*** Narrative description of observed changes.	Description of changes in the institutional environment at the facilities after the intervention compared with before the intervention, considering behavioral determination elements.

### Primary outcomes

The primary outcome was defined by two variables, calculated as indicators: 1) Provider practiced breastfeeding-friendly *clinical* behaviors, and 2) Provider practiced breastfeeding-friendly *interpersonal* behaviors. Clinical behavioral indicators focused on the provision and content of breastfeeding counseling, or the “what” of counseling, while interpersonal behavioral indicators assessed relational skills, capturing the “how” of their interactions. The frequency of each of the nine clinical behaviors was determined by the percentage of “yes” responses among all responses, which resulted in a continuous variable ranging from 0% to 100%. This was then combined and adjusted for clustering by staff with facility as strata variable, to arrive at a mean breastfeeding-friendly clinical behavior indicator. For the three interpersonal behaviors, frequency for each was assessed using a five-point Likert scale, where responses of “a lot” or “a great deal” were categorized as positive (“yes”), while “moderate amount” or lower was treated as a “no” response to account for potential courtesy bias, as observed in previous research validating interpersonal aspects of family planning counseling [59–62]. These results were then combined and adjusted for clustering by provider with facility as strata variable, to arrive at a mean breastfeeding-friendly interpersonal behavior indicator.

### Secondary outcomes

The objectives of these secondary outcome indicators were to assess if and how the intervention facilitated the adoption of breastfeeding-friendly practices by providers. The secondary outcomes were selected based on formative research and the theory of change, each reflecting key behavioral determinants: maternal experience captured weaknesses in interpersonal counselling; provider knowledge and attitudes reflected underlying beliefs and gaps; and institutional measures addressed leadership, tools, and role clarity. Together, these outcomes aligned with the targeted determinants and assessed whether the intervention was shifting individual and institutional drivers of behavior. We explored maternal experience of care through changes in validation agreement rates (the frequency of the same response by mother and observer report) after versus before intervention, and measuring mothers’ feelings about breastfeeding after versus before intervention. We also explored changes in providers’ knowledge and attitudes towards breastfeeding based on a range of interviewer- and self-assessments. Finally, we documented any changes (or not) in the institutional environment at the study facilities under two thematic behavioral determinant clusters; psychosocial and environmental.

### Sample size

To calculate the primary outcome of interest—breastfeeding friendly clinical and interpersonal practices during routine consultations—there was limited to no existing data on current frequency. However, discussions with local stakeholders and a review of a report on nutrition counseling practices among primary care providers in Kosovo [63] suggested that an average frequency rate of 25% for all indicators was a reasonable assumption. Using moderate to high sensitivity (≥65%) and specificity (≥75%), along with a ± 7% precision and α = 0.05 (assuming a normal approximation to the binomial distribution), the required sample size for primary outcome indicators was determined to be 255 at baseline and endline each (total n = 510), calculated based on Buderer’s formula [64]. During the data collection period, all mothers attending infant consultations were invited to participate until the minimum sample size for each site was achieved.

### Data analysis

Stata17 [65] was used to analyze the primary outcomes data on provider behaviors (clinical and interpersonal). The analysis assessed breastfeeding-friendly practices during routine consultations according to observer reports and mother reports before and after the intervention as the proportion of specified behaviors occurring during the consultation. The primary outcome was analyzed using linear regression. Individual behaviors were treated as a binary variable and analyzed using generalized linear models (link: identity, family: binomial). To account for the non-independence of observations within providers and potential variability across health facilities, all primary outcome analyses were adjusted for clustering at the provider level using robust standard errors, with facility specified as a stratification variable. This approach corrects standard errors for repeated observations of individual providers and controls for unmeasured facility-level characteristics that could influence provider behavior. Two of the five municipalities had two facilities represented in the sample; in these cases, data from the facilities were combined to represent a single facility per municipality. All “don’t know” responses excluded.

For the secondary outcome indicator measuring maternal experience of care, agreement rates (the level of consistency between two sets of measurements, in this case observer and mother reports) was calculated for each breastfeeding-friendly indicator in two-by-two tables, with all “don’t know” responses excluded from the matched pairs analysis. Cohen’s kappa [66] values were calculated to assess interrater agreement, where 1 indicates complete agreement, 0 no agreement or independence and -1 perfect disagreement [67]. Provider and mother rankings of influencers on the infant feeding decision were compared by calculating the difference in the combined top two rankings. Qualitative data, including assessment of the institutional environment, was categorized into thematic groupings of observed and reported barriers and facilitators to improving breastfeeding-friendly practices. It was then analyzed using the BCD framework to map behavioral determinants at individual and institutional levels across psychosocial and environment dimensions. The analysis focused on changes in behavioral determinants following the intervention and their influence on the targeted behaviors, to assess relevance, effectiveness, and potential synergistic effects.

### Ethical approval and consent

Ethical approval was granted by the Hospital and University Clinical Services of Kosovo Professional Ethics Committee (#133/6240), the Kosovo Doctors Chamber, LSHTM Observational and Interventions Research Ethics Committee (#16357) and Johns Hopkins School of Public Health Institutional Review Board (#9653). Written informed consent was obtained from all participants (mothers and providers) prior to observations during the baseline data collection and changed to verbal informed consent during the endline data collection to comply with COVID-19 safety protocols. The consent rate was 100% from providers and 98% among mothers (2% decline at exit interview stage).

## Results

### Sample description

Sample characteristics for the observation of 72 healthcare providers across 609 consultations, (277 at baseline and 332 at endline) are depicted in Table 3. Two-thirds of consultations were conducted by nurses, and over 90% involved female providers. Nearly all mothers identified as ethnically Albanian, with 84% breastfeeding previous child(ren). Fewer than half (45%) of mothers’ current infant were being exclusively breastfed. The primary reason for just over half of all consultations was infant immunization, as FMCs are the only authorized sources of infant vaccinations in Kosovo, where coverage rates are high [48]. We interviewed 117 providers (36 at baseline and 81 at endline); 57% were nurses, 93% female and the average age was 52 years old (68% of providers were aged 50 years or older).

**Table 3:** Sample characteristics of paired observations and exit interviews. Provider and maternal demographic and consultation characteristics at baseline and endline.

Observations and exit interviews				
**CHARACTERISTICS: INFANT**	**Baseline %** **n = 277**	**Endline %** **n = 332**	**Combined n = 609**	**Combined %**
**Consultation type**				
Immunization	52%	53%	320	53%
Routine check for baby	16%	25%	126	21%
Acute visit for baby	32%	21%	158	26%
Acute visit for mother	0%	1%	5	1%
**Infant age**				
<1 month	27%	20%	141	23%
1-3 months	29%	31%	183	30%
4-6 months	13%	20%	101	17%
7-12 months	31%	29%	184	30%
**Current breastfeeding status**				
Exclusive (<6 months)	39%	50%	274	45%
Any (0–12 months)	38%	23%	178	29%
No, but previously did (0–12 months)	14%	15%	87	14%
Never (0–12 months)	9%	13%	70	11%
**CHARACTERISTICS: MOTHER**	**%**	**%**	**n = 609**	**%**
**Mother’s ethnicity**				
Albanian	95%	95%	581	95%
Did not answer	5%	5%	28	5%
**Mother’s education level**				
Primary or pre-primary	16%	10%	78	13%
Secondary	50%	43%	281	46%
Higher	31%	46%	239	39%
Did not answer	3%	1%	11	2%
**Current age of mother**				
18-24	20%	22%	127	21%
25-29	40%	31%	216	35%
30-34	24%	33%	175	29%
35+	16%	13%	87	14%
Did not answer	0%	1%	4	1%
**Prior breastfeeding counseling**				
During pregnancy	39%	41%	244	40%
During the first two days after baby’s birth	65%	80%	448	74%
Anytime within the first month of baby’s birth (3–30 days after birth)	61%	76%	421	69%
**Mother’s prior parity**				
0	37%	40%	236	39%
1 or more	63%	60%	372	61%
*Mother did breastfeed prior child/ren*	52%	51%	*312*	*84%*
*Mother did not breastfeed prior child/ren*	11%	9%	*59*	*16%*
Did not answer	0%	0%	1	0%
**CHARACTERISTICS: PROVIDER**	**%**	**%**	**n = 609**	**%**
**Provider position**				
Consultations with a Nurse	67%	63%	394	65%
Consultations with a Doctor	33%	37%	215	35%
**Provider gender**				
Consultations with a Female provider	90%	95%	562	92%
Consultations with a Male provider	10%	5%	47	8%

Chi-squared tests comparing sample characteristics across the two data collection periods showed variation in three areas: at endline, 76% of mothers reported receiving breastfeeding counseling within the first month after birth, compared to 61% at baseline (χ² p < 0.001). Acute care visits accounted for a lower proportion of all visits at endline (21%) than at baseline (32%) (χ² p = 0.007). Due to COVID-19 prevention measures, only 36% of mothers were accompanied by a companion during consultations at endline, down from 80% at baseline (χ² p < 0.001).

### Primary outcome indicators

#### Clinical and interpersonal behaviors.

Associations between clinical and interpersonal behaviors and outcomes are presented in Table 4, with the difference in “after intervention” versus “before intervention”. The intervention was associated with an improvement in provider practices. There was an adjusted 14.9 percentage point increase in the mean frequency of *clinical* behaviors after versus before the intervention (95% CI: 6.4-23.4). The SD increased from 17.0 at baseline to 26.1 at endline. This aggregate indicator captures the mean frequency of nine clinical practices, as reported by mothers during exit interviews. Notable changes among individual clinical indicators included a: 39.0-point increase in “Providers explained a woman’s physiological ability to breastfeed” (95% CI: 26.4-51.6); 21.8-point increase in “Providers asked mothers if she had any breastfeeding questions or concerns” (95% CI: 8.2-35.4); and 18.7-point increase in “Providers explained follow-up visits required” (95% CI: 6.0-31.3). More modest increases were observed for “Giving take-home materials” (+11.9 points, 95% CI: 7.5-16.3), “Explaining support resources available” (+11.8 points, 95% CI: 5.0-18.7), and “Observing breastfeeding” (+12.1 points, 95% CI: 6.5-17.8). One indicator, “Not promoting breastmilk substitutes”, remained stable at an already high baseline level (98% at baseline and endline).

**Table 4:** Changes in the frequency of breastfeeding-friendly provider behaviors during routine consultations with infants aged 0-12 months – after versus before intervention.

	BEFORE			AFTER			DIFFERENCE^1^
**INDICATOR/BEHAVIOR**	**N**	**Frequency**	**Std Dev**	**N**	**Frequency**	**Std Dev**	**Adjusted Difference % pt (95% CI** ^**2**^ **)**
**Provider practiced breastfeeding-friendly CLINICAL BEHAVIORS (mean)**	**230**	**28.2%**	**17.0**	**314**	**43.5%**	**26.1**	**14.9 (6.4-23.4)**
*Frequency of individual clinical behaviors:*							
Provider explained the benefits of breastfeeding	262	55.0%	49.8	329	60.8%	48.9	8.9 (-6.1-23.9)
Provider explained a woman’s physiological ability to breastfeed	250	9.2%	29.0	324	51.5%	50.1	39.0 (26.4-51.6)
Provider asked mother if she had any breastfeeding questions or concerns	252	21.8%	41.4	324	46.0%	49.9	21.8 (8.2-35.4)
Provider explained follow-up visits required	269	58.0%	49.4	327	78.6%	41.1	18.7 (6.0-31.3)
Provider inquired about mothers’ support structure	246	5.3%	22.4	319	6.0%	23.7	3.1 (-3.8-10.1)
Provider gave take-home material about breastfeeding	251	1.6%	12.5	320	15.3%	36.0	11.9 (7.5-16.3)
Provider explained breastfeeding support resources available	252	6.0%	23.7	317	22.4%	41.8	11.8 (5.0-18.7)
Provider did not promote breastmilk substitutes (formula)	246	98.0%	14.1	321	98.1%	13.6	-0.1 (-2.8-1.6)
Provider observed mother breastfeeding	246	5.7%	23.3	324	20.7%	40.6	12.1 (6.5-17.8)
**Provider practiced breastfeeding-friendly INTERPERSONAL BEHAVIORS**^**3**^ **(mean)**	**263**	**55.9%**	**39.5**	**329**	**69.2%**	**37.0**	**13.3 (5.2-21.3)**
*Frequency of individual interpersonal behaviors:*							
Provider really listened to, and understood the concerns of, mother.	266	66.5%	47.3	331	80.7%	39.6	14.4 (6.9-22.1)
Provider made mother feel comfortable to express her opinions, feelings and concerns.	266	66.5%	47.2	329	79.0%	40.8	12.9 (4.8-20.9)
Provider explained things well and gave practical help in a way mother could understand	263	34.2%	47.5	331	47.7%	50.0	12.0 (-1.0-25.1)

^1^Adjusted for clustering by staff, with facility as strata variable. ^2^CI = Confidence Interval. ^3^Likert scale converted to binary result by collating “A great deal” or “A lot” into “Yes” and “Not at all”, “A little”, “A moderate amount” to “No”.

Interpersonal practices also improved meaningfully, with an adjusted 13.3 percentage points increase in the mean frequency of *interpersonal* behaviors after versus before the intervention (95% CI: 5.2-21.3). This included improvements in “Providers listening and understanding mothers’ concerns” (+14.4 points, 95% CI: 6.9-22.1), “Making mothers feel comfortable expressing opinions and feelings” (+12.9 points, 95% CI: 4.8-20.9), and “Explaining things clearly and providing practical help” (+12.0 points, 95% CI: -1.0-25.1). The mean standard deviation for interpersonal practices remained high (39.5 at baseline and 37.0 at endline). Six indicators recorded a “don’t know” response of greater than 5%, but none exceeded 7%.

### Secondary outcome indicators

#### Maternal experience of care.

Maternal rating of overall experience at the facility improved one point on the five-point Likert scale, from “average” to “good” after versus before the intervention. Similarly, their agreement with the statement “I believe I can successfully breastfeed my baby” improved one point from “moderate” to “a lot” after versus before the intervention. Two of the three indicators measuring maternal report in the difference in feelings about breastfeeding after their consultation improved one point on the five-point Likert scale, from “little” to “moderate”; “Value of breastfeeding for my baby’s health” and “Length of time I will breastfeed baby”. There was no change in the third indicator, “My ability to overcome breastfeeding challenges”. See S6 File for a detailed table of results. Table 5 compares maternal report with direct observation regarding the frequency of breastfeeding-friendly behaviors during routine consultations with infants aged 0–12 months. Mean frequency of clinical and interpersonal behaviors is presented (with SD), alongside kappa and matched pairs, however the agreement rates is not presented because these indicators reflect averages across multiple items where exact agreement is inherently less likely and standard agreement metrics would misrepresent the underlying variation.

**Table 5:** Evaluating maternal report against direct observation of the frequency of breastfeeding-friendly behaviors during routine consultations with infants aged 0-12 months – a comparison of results before and after intervention.

	BEFORE INTERVENTION					AFTER INTERVENTION				
INDICATOR/ BEHAVIOR	MOTHER Frequency (95% CI)	OBSERVER Frequency (95% CI)	Agreement	Cohen’s kappa (k)^1^	Matched Pairs (N)	MOTHER Frequency (95% CI)	OBSERVER Frequency (95% CI)	Agreement	Cohen’s kappa (k)^1^	Matched Pairs (N)
Provider practiced breastfeeding-friendly CLINICAL behaviors, mean | SD^2^	28.2% SD: 17.0	38.8% SD: 18.1	..	0.09	187	43.7% SD: 25.8	51.7% SD: 21.8	..	0.14	249
*Frequency of individual clinical behaviors:*										
Provider explained the benefits of breastfeeding.	55.0% (49.0-61.0)	60.7% (54.7-66.7)	69%	0.37	245	60.8% (55.5-66.1)	58.1% (52.7-63.5)	75%	0.47	324
Providers explained a woman’s ability to breastfeed.	9.2% (5.6-12.8)	36.5% (30.5-42.5)	62%	0.07	229	51.5% (46.1-57.0)	40.8% (35.5-46.1)	71%	0.43	323
Providers asked mother if had questions	21.8% (16.7-27.0)	36.2% (30.1-42.2)	68%	0.28	225	46.0% (40.5-51.4)	66.8% (61.2-72.4)	73%	0.46	270
Provider explained follow-up visits required.	58.0% (52.1-63.9)	93.8% (91.0-96.7)	59%	0.04	267	78.6% (74.1-83.1)	95.7% (93.5-97.9)	83%	0.24	319
Provider inquired about mothers’ support structure.	5.3% (2.5-9-8.1)	13.1% (8.9-17.4)	83%	0.07	224	6.0% (3.3-8.6)	16.6% (12.6-20.6)	82%	0.14	318
Provider gave take-home material about breastfeeding.	1.6% (0.0-3.1)	5.1% (2.4-7.8)	93%	-0.03	232	15.3% (11.3-19.3)	16.4% (12.4-20.4)	89%	0.60	318
Provider explained breastfeeding support resources available.	6.0% (3.0-8.9)	5.9% (3.0-8.8)	88%	-0.06	230	22.4% (17.8-27.0)	26.2% (21.4-31.0)	80%	0.47	313
Provider did not promote breastmilk substitutes (formula)	98.0% (96.2-99.7)	98.8% (97.4-100.2)	96%	-0.02	221	98.1% (96.6-99.6)	93.1% (90.3-95.8)	92%	0.04	320
Provider observed mother breastfeeding.	5.7% (2.8-86.1)	11.4% (7.5-15.3)	86%	0.18	235	20.7% (16.2-25.1)	16.9% (12.8-21.0)	92%	0.75	318
**Provider practiced breastfeeding-friendly INTERPERSONAL behaviors** ^**3**^ **, mean | SD**	**53.4%** **SD: 40.1**	**13.6%** **SD: 27.4**	**..**	**0.02**	**246**	**68.8%** **SD: 37.2**	**53.6%** **SD: 41.4**	**..**	**0.29**	**329**
*Frequency of individual interpersonal behaviors:*										
Provider really listened to, and understood the concerns of, mother.	66.5% (60.8-72.2)	14.3% (10.1-18.5)	38%	0.01	255	80.7% (76.4-84.9)	57.8% (52.5-63.2)	65%	0.23	331
Provider made mother feel comfortable to express opinions, feelings.	66.5% (60.8-72.2)	14.7% (10.4-18.9)	38%	0.00	255	79.0% (74.6-83.4)	58.1% (52.8-63.5)	66%	0.26	329
Provider explained things well and gave practical help in a way mother could understand.	34.2% (28.4-40.0)	11.9% (8.0-15.8)	68%	0.15	254	47.7% (42.3-53.1)	44.9% (39.5-50.3)	75%	0.49	331

^1^Unweighted. ^2^SD = Standard Deviation. ^3^Likert scale converted to binary result by collating “A great deal” and “A lot” into “Yes” and “Not at all” and “A little” into “No”. Responses indicating “A moderate amount” or Don’t Know were excluded.

***Clinical indicators:*** Before the intervention, observers’ reported frequency for all but one clinical indicator (related to breastfeeding support resources), was higher than maternal report. The observer overreporting difference across the remaining eight clinical indicators ranged from between 0.8 and 38.5 percentage points. The mean reported frequency of clinical provider behaviors by mothers and observers before the intervention was 28.2% and 38.8% respectively, while after the intervention it was 43.7% and 51.7% respectively. This indicates an overall reduction in the gap in agreement rates between mother and observer reports, due to six of the nine clinical indicators (in particular “Provider explained follow-up visits required”, -24 points). The other three clinical indicators recorded a widening in the agreement gap of between 1 and 8 percentage points. Standard deviations increased at endline versus baseline for both mother (17.0 to 25.8) and observer (18.1 to 21.8) reports.

***Interpersonal indicators:*** Observer overreport of the frequency of interpersonal indicators compared with maternal report was more significant than for clinical indicators both before and after intervention. Mothers reported an average frequency rate of 53.4% versus observers at 68.8% before the intervention. However, agreement rates after the intervention came together by between 7 and 28 points for the three interpersonal behaviors.

This reduction in the agreement gap was due to the much larger frequency rate increase recorded by observers (an average of 40 points) versus mothers (an average of 15.4 points) after the intervention. The interpersonal indicator that experienced the least change in agreement rates (7 points) was “Provider explained things well and gave practical help in a way mother could understand”, given it recorded a lower frequency rate by mothers compared with the other two interpersonal indicators before the intervention. The standard deviation for maternal reports remained high before and after the intervention (40.1 to 37.2), while observer SDs increased from 27.4 to 41.4.

Cohen’s kappa analysis generally indicated improved agreement between observer and maternal reports after versus before the intervention, strengthening confidence in reported changes. Before the intervention, most clinical indicators had only slight agreement (κ ≤ 0.20), with some even showing poor agreement (κ < 0). However, these low values likely reflect skewed marginal distributions due to the dominance of one category (e.g., most say “No”) or low prevalence of certain behaviors (e.g., “observed breastfeeding”), both of which are known to affect kappa estimates. After the intervention, kappa rates increased for mean clinical (κ = 0.14 versus κ = 0.09) and interpersonal (κ = 0.09 versus κ = 0.29) indicators. Several indicators improved into the fair to moderate agreement range, with some indicators reaching substantial agreement, e.g., observing breastfeeding (κ = 0.75) and providing take-home materials (κ = 0.60).

### Providers’ knowledge and attitudes

Overall, there were notable changes in provider knowledge, attitude and self-efficacy after versus before the intervention, with details provided in S6 File. Provider knowledge across 13 indicators increased 16.5 percentage points after versus before the intervention period. This means that 96% of answers given by providers in response to interview questions related to their knowledge about breastfeeding were correct after the intervention versus 80% being correct before the intervention. Provider attitude across 13 indicators increased 12.8 percentage points after versus before the intervention period. This means that 84% of answers given by providers in response to interview questions related to their attitude towards breastfeeding were positive after the intervention versus 71% being positive before the intervention.

Provider self-efficacy across 13 indicators showed varying rates of improvement and decline after the intervention compared to before, based on an ‘ideal response’, meaning “agree/strongly agree” or “disagree/strongly disagree” depending on the question. Seven of the indicators recorded a higher number of ideal responses after versus before the intervention, for a cumulative 16 percentage point increase in the rate of ideal responses. Eight of the indicators recorded a higher number of unideal responses after versus before the intervention, for a cumulative 9 percentage point increase in the rate of unideal responses. Provider opinion on who influenced maternal infant feeding decision shifted after versus before the intervention, moving to more closely align with maternal opinion. Provider opinion of the influence of family (infant’s grandmother or father) changed substantially after versus before the intervention, with only 15% of providers ranking them as first or second choice at baseline versus 52% at endline, although this still represents a gap of 37 points compared to the maternal report combined tally of 89% (only collected at baseline).

### Institutional environment

To better understand the contextual factors that enabled or constrained the adoption of breastfeeding-friendly provider practices, we assessed changes in the institutional environment across participating facilities. Drawing on behavioral science constructs, we examined shifts across key organizational determinants—including governance, leadership, training, norms, infrastructure, and feedback mechanisms—before and after the intervention. Table 6 summarizes these institutional-level findings, highlighting both areas of progress and remaining gaps in system-level support for sustained behavior change.

**Table 6:** Changes in the institutional environment influencing breastfeeding-friendly practices in primary care. Observed changes in governance, leadership, training, norms, infrastructure, and social environment before and after the intervention.

Behavioral Determinant	Before Intervention	After Intervention
Political will and resources (leadership, prioritization, policy)	Breastfeeding was not a visible priority at the national or facility level. No written policy or guidance existed for primary care. Structural reforms disrupted services and created confusion about roles.	A national breastfeeding pledge for primary care was developed and endorsed by MOH. Facility and national leaders participated in the study, but there was no evidence of institutionalization or long-term budget to maintain gains or scale-up activities.
Facility leadership and roles (governance, norms, rewards)	No breastfeeding or infant feeding coordinators were in place. Breastfeeding support was absent from job descriptions.	Two peer-nominated coordinators led activities at each facility, improving visibility and value, though positions were not officially recognized. No new job descriptions.
Education and training (knowledge, skills)	Most providers had not received formal breastfeeding training in over a decade, but had adequate knowledge.	Providers received virtual skills training certified by Kosovo’s Doctors and Nurses Chambers. There was an increase in provides’ knowledge and attitude scores.
Organizational culture, norms and routines (motives, rewards, habits)	Provider behaviors were limited to minimal counseling, even when prompted by patient records. There was no encouragement of breastfeeding during immunization. No systems existed to monitor breastfeeding counseling practices or provide feedback to providers.	Observed increases in breastfeeding-friendly behaviors and more positive attitudes. UNICEF intention to add the protocol of encouraging mothers to breastfeed during immunization in future training, but no immediate provider behavior change was seen in this behavior. No new supervision mechanisms were introduced during the intervention period.
Physical environment (tools, cues, infrastructure)	Facilities lacked basic tools - no brochures and outdated posters. Only one facility had a private space suitable for breastfeeding counseling.	New brochures and posters were distributed to all facilities. A new counseling room was opened in one of the study sites in the year following implementation.
Social environment (influencers, relationships, networks)	Providers underestimated the role of family in influencing mothers. Power imbalances between doctors and nurses contributed to confusion over who should lead breastfeeding counseling.	Providers’ understanding of family influence improved substantially. However, clarity on counseling roles between cadres remained limited likely due in part to the ongoing restructuring of primary care and political instability.

There were visible improvements in the domains of political will and facility leadership/roles with a national breastfeeding pledge for primary care and peer-nominated breastfeeding coordinator positions created. However, there was not opportunity (given time and leadership will) for institutionalization of these gains during the intervention period, such as moving from a pledge to policy and changing job descriptions. Providers received updated training and tools (posters, brochures) to support breastfeeding counseling and there were observed increases in breastfeeding-friendly knowledge, attitudes and practices, but again critical system elements that might support institutionalization of these gains, such as new monitoring mechanisms and formal inter-cadre role clarity were not realized.

Additional shifts, such as UNICEF’s intention following the intervention to increase the breastfeeding-friendly aspects of its national immunization training protocols and pilot Home Visiting Program (which the MOH has since scaled to 30 municipalities [68]), as well as providers’ recognition of the importance of engaging family to support breastfeeding, are promising for longer term behavior changes.

## Discussion

### Key results

This before-and-after evaluation assessed whether a behaviorally informed intervention could improve breastfeeding-friendly practices during routine consultations in a primary healthcare setting in Kosovo. The intervention was associated with meaningful improvements in both clinical and interpersonal behaviors. The greatest increases were observed in behaviors that supported maternal confidence, such as providers explaining mother’s physiological ability to breastfeed. These findings contribute to growing evidence that provider behavior change interventions must extend beyond knowledge transfer. By tapping into underlying motives, such as *status*, *nurture*, and *affiliation*, and leveraging those in emotionally resonant activities like Emo-Demo videos, the intervention achieved measurable shifts in breastfeeding-friendly behaviors. The use of BCD enabled the targeting of deep behavioral drivers, not just surface-level barriers.

Notably, the gap between maternal and observer reports of provider behaviors narrowed. At baseline, observers consistently rated provider performance higher than mothers did, a pattern observed in prior studies across health domains and geographies. For example, studies on family planning and maternal care in Kenya, Haiti, and Malawi have documented notable discrepancies between provider-reported or observed care and women’s self-reported experiences, especially regarding counseling, consent, and respectful communication [59,61,62]. One explanation might be that providers were not using simple, accessible language, which could have affected maternal understanding of the counseling content. In contrast, the observers in this study, senior clinicians themselves, were more likely to interpret provider communication as intended. This highlights how the experience of care can sometimes diverge from what is technically delivered.

After the intervention, we observed improved alignment between observer and maternal report, with narrowing agreement gaps across several indicators. This suggests the intervention helped move provider actions towards being more client-centered, reflecting a better understanding of how to counsel mothers in a more effective way. Similar outcomes were found in interventions in Tanzania and Ghana, where shifts in client-provider communication and mutual understanding were attributed to emotionally engaging, co-created solutions that redefined professional norms [31,42]. Mothers are the ultimate arbiters of care quality, particularly for behaviors like breastfeeding, where trust and emotional support strongly influence continuation. The confidence intervals observed across outcome measures further support the conclusion that the intervention was effective, albeit with some variability in precision. The relatively narrow and positive CIs for the primary outcome indicators indicate robust improvements unlikely to be due to chance.

The improvements observed across clinical and interpersonal practices (primary outcomes), combined with changes in maternal experience, provider knowledge and attitudes, and institutional environment (secondary outcomes), align closely with the barriers and facilitators the intervention was designed to address. The Theory of Change (Fig 1) and Logic Model (S1 File) make this linkage explicit, showing how activities targeted specific BCD determinants and how several barriers were reframed through HCD methods into feasible strategies. This alignment between theory, design, implementation, and outcomes underscore the value of grounding evaluation measures in a clear behavioral theory of change.

### Limitations

The intervention and endline evaluation took place during the COVID-19 pandemic, which introduced health system disruptions, limited in-person engagements, and may have influenced provider and maternal behaviors during endline data collection. Data collectors at endline noted signs of COVID-related apathy among providers, attributed to increased workload, staffing shortages, and shifting responsibilities. While it is plausible that the intervention helped counteract some of these negative pressures and observed improvements in counseling behaviors might have been otherwise larger, the extent of this cannot be measured with certainty. Additionally, some intervention components, such as the facility breastfeeding pledge and clinical support app, were only partially implemented. As a result, it is difficult to assess whether the full intervention package functioned synergistically. Although the intervention yielded positive outcomes, it was not possible to isolate the effects of individual activities, determine which motivational drivers were most influential, or assess whether the improvements were primarily due to the synergistic effect of the combined activities. However, provider interviews at endline offered insight into which activities were most impactful. Nearly all respondents recalled exposure to multiple components, and when asked to identify the most helpful, brochures, virtual skills training, Emo-Demo videos, and posters were cited most frequently. Providers indicated that these activities refreshed existing information, boosted their confidence, and provided simple, engaging tools that could be shared with mothers and their families.

While the primary outcomes showed statistically significant improvements with reasonably narrow confidence intervals, some individual behaviors exhibited wider CIs or intervals that crossed zero. This suggests greater uncertainty around improvements in less frequent or more complex provider behaviors, and highlights the need for cautious interpretation of these specific results. To quantify alignment between maternal and observer reports, Cohen’s kappa was calculated for each indicator. While kappa values remained within slight to moderate ranges, they should be interpreted with caution. In cases where the prevalence of a behavior is low or when one response dominates (e.g., “no”), κ values are known to be deflated. Furthermore, there is no universally accepted magnitude guidelines [67]. Therefore, the narrowing of raw agreement gaps (for the prevalence of the 12 clinical and interpersonal behaviors) and observed improvements in maternal perceptions suggest progress, even if κ values appear weak. Given that several indicators capture subjective experiences, it is plausible that maternal interpretations of the interaction shaped their reports more than the observable content, an interpretation supported by findings from other studies [59,61,69].

These limitations also have implications beyond this study, particularly for how behavior change interventions are evaluated when provider practices are measured indirectly. Using maternal exit interviews as a proxy for provider behavior brings a particular interpretive challenge. If mothers report that breastfeeding-friendly counselling occurred, but the observed fidelity was low, this may overestimate the true extent of behavior change. Conversely, if observers report breastfeeding-friendly counselling delivered but mothers do not, this may underestimate the intervention’s effect. In either case, interpretation of outcome effects becomes less certain: a lack of change in exit interview data could reflect measurement error rather than poor delivery, while a positive change could mask gaps in fidelity. This is a common issue in evaluating provider behavior change where direct observation is resource-intensive. The implication is that outcome data should be interpreted alongside robust process and fidelity measures, so there is the ability to distinguish between a weak intervention, weak delivery, and weak measurement.

The study’s before-and-after design presents limitations. Without a comparison group, it was not possible to attribute changes solely to the intervention. Additionally, the individual mothers, and in some cases providers, surveyed before and after the intervention were not the same, but the samples were comparable in their key characteristics across both time points according to the chi-square test performed. There was potential for observer bias and the Hawthorne effect (normative behavior due to being observed) during consultation observation, although these risks were reduced through the use of a structured WHO/UNICEF-adapted checklist and rigorous data collector training. Similarly, maternal reports, a primary outcome measure and strength of the study, may have been affected by courtesy bias (i.e., answering politely) and/or recall bias (i.e., the mother might not have had a chance to process all the messaging she just received). A similar issue was hypothesized in a study validating family planning quality of care in Ecuador, Uganda and Zimbabwe [20]. Finally, the research team’s involvement in all phases of the intervention (design, implementation, and evaluation) introduced the potential for bias, which was mitigated through detailed process documentation, reflexivity, and transparent reporting.

### Interpretation

Despite these limitations, the intervention’s observed effects are encouraging. Improvements in breastfeeding counseling were not only reflected in behavior change but also in how mothers experienced care. The narrowing of the gap between observer and maternal reports suggests that providers internalized new behaviors and applied them more effectively. Despite improvements in breastfeeding-friendly practices, institutional changes lagged. Tools, training, facility-level pledges and peer leadership roles were introduced, but were not embedded into supervision, job descriptions, or broader policy. Sustaining and scaling this approach in fragile health systems requires embedding behavioral gains into existing institutional structures. In Kosovo, change was constrained because new practices remained outside core accountability mechanisms. Key levers for institutionalization include integrating breastfeeding counselling into supervision checklists and performance reviews, incorporating new roles into formal job descriptions, aligning with budgeted policies, and embedding tools such as apps and job aids into national health information systems. Without these reinforcements, provider-level improvements risk erosion over time, but with them, emotionally engaging, provider-centered activities can be sustained and scaled within fragile primary care systems.

Overall, within the theory of change, the improved agreement between observer and maternal report suggests that the intervention effectively disrupted the existing behavioral setting and activated both motivated and reactive mechanisms, enabling providers to adopt breastfeeding-friendly practices more consistently and visibly. While Cohen’s kappa values were limited in some areas due to methodological constraints, the overall direction of change strengthens confidence that mothers experienced care that was both more behaviorally meaningful and more technically aligned with breastfeeding-friendly practices.

The observed standard deviations (SDs) in clinical and interpersonal behaviors warrant review. The moderate SD for clinical behaviors suggests some inconsistency in breastfeeding-friendly practices, and while the increase in SD at endline indicates that the variation grew alongside frequency rates, this could reflect differing rates of uptake. In contrast, the persistently high SD for interpersonal behaviors indicates greater variability in *how* providers engaged with mothers. This variability is not unexpected, given that interpersonal skills are greatly influenced by individual provider personality and are therefore typically harder to shift uniformly [29,70]. Behavioral science underscores the need for interventions to couple interpersonal training with reinforcement activities that target organizational norms in support of patient-centered care to achieve more consistent improvements, as echoed in several reviews [29,37,41,47].

### Generalizability

The findings offer important insights for other low-resource or fragile settings, but generalizability is constrained by the study design and context. The relatively small sample of observed providers, particularly in two municipalities, and the distinct sociopolitical and economic conditions in Kosovo limit direct application elsewhere. Still, the intervention’s structure, use of behavioral science, and emphasis on participatory design are broadly applicable. Future studies in diverse settings are needed to confirm whether such a behaviorally informed approach can reliably improve provider practices and service experience at scale.

## Conclusions

This study demonstrates that a behavioral science grounded, co-designed intervention can meaningfully improve primary healthcare provider behaviors in support of breastfeeding, even within the constraints of a fragile health system and global pandemic. Improvements were observed in both clinical and interpersonal provider practices, well-established contributors to increased breastfeeding rates. Notable variability across providers remained, especially in interpersonal behaviors, which highlights the importance of strategies that foster shared norms and consistent expectations for patient-centered care. Institutional change was more limited; while tools and peer roles were introduced, they were not embedded into supervision or policy, leaving improvements vulnerable to system pressures. Still, the findings offer practical insights into how behavioral science-grounded, co-designed approaches can strengthen provider practice. Sustaining gains in breastfeeding-friendly practice will likely require deeper integration into Kosovo’s primary health care system.

## Supporting information

S1 File Logic model. (DOCX)

S2 File Observation scoring guide. (DOCX)

S3 File Observation checklist – English and Albanian. (DOCX)

S4 File Client exit interview questionnaire – English and Albanian. (DOCX)

S5 File Provider interview questionnaire. (DOCX)

S6 File Secondary outcome indicators – detailed tables. (DOCX)

S1 Checklist Inclusivity in global research. (DOCX)
