# Supplementary material for: Can behavioral science advance breastfeeding-friendly primary care? Key findings from an evaluation in Kosovo
Source: PLOS Glob Public Health. 2025 Oct 31;5(10):e0005276. doi: 10.1371/journal.pgph.0005276 (PMC12578251; doi:10.1371/journal.pgph.0005276)
Supplement: S1 File — (DOCX) [file pgph.0005276.s001.docx]

**S1 File. Logic model**

| **Barriers / Facilitators (formative research)** | **Intervention Components** | **BCD Behavioural Domains Targeted** | **Intended**  **Outcomes** |
| --- | --- | --- | --- |
| Weak interpersonal counselling skills; lack of standardised tools | Virtual skills training; Clinical support app | Capability (knowledge, scripts); Performance support; Routines | Providers deliver consistent, effective breastfeeding counselling |
| Knowledge gaps in specific domains (prenatal counselling, formula use, vaccination, recognising problems) | Virtual skills training; Clinical support app | Executive (knowledge, skills); Motivated (self-efficacy/confidence) | Providers equipped to give accurate, evidence-based advice across contexts |
| Limited time for counselling during routine consultations | Integration of counselling into routine workflows (reinforced through Emo-Demo videos, app, and training) | Reactive (habits, scripts); Physical (time/resources); Social (norms about prioritisation) | Breastfeeding support embedded into routine service delivery |
| Resistance/difficulty in changing entrenched habits and routines | Transformational Forums; Integration of counselling into routine workflows; Motivational video | Reactive (habits, routines); Motivated (re-evaluation, peer influence) | Providers adopt new routines and normalise breastfeeding counselling |
| Confusion over provider roles; unclear accountability | Breastfeeding Coordinator; Facility pledge | Social (roles, norms); Motivated (status, affiliation) | Clear responsibility and peer recognition for breastfeeding support |
| Low provider motivation; perception of breastfeeding as low priority | Transformational Forums/video; Emo-Demo videos; PhotoVoice activity | Motivated (status, affiliation, nurture, re-evaluation) | Providers see breastfeeding as integral to professional identity and quality care |
| Lack of provider feedback or recognition for impact | Transformational Forums; Motivational video; Facility pledge | Motivated (rewards, incentives, pride, peer recognition) | Providers experience social validation and reinforcement for breastfeeding support |
| Limited leadership engagement; weak institutional supports (policies, training, infrastructure, governance, supervision) | Advocacy with facility/MOH leaders; Facility pledge; Breastfeeding Coordinator | Social (governance, leadership, institutional norms); Motivated (role-modelling); Physical (infrastructure, resources) | Leaders actively endorse and sustain breastfeeding-friendly practices; institutionalisation initiated. |
| Absence of visual / environmental cues supporting breastfeeding | Poster and brochure | Physical (cues, materials); Social (norm reinforcement) | Breastfeeding support becomes a visible and expected part of care |
| Providers failing to engage family influencers (fathers, grandmothers) despite their strong role in feeding decisions | PhotoVoice activity; Emo-Demo videos; Poster and brochure | Social (family influence, norms); Motivated (emotional engagement) | Providers and families aligned; fathers/grandmothers recognised as partners in breastfeeding support |
| Infrastructure barriers (e.g., lack of counselling rooms) | Advocacy; Facility pledge (as interim step towards policy change) | Physical (infrastructure, space); Social (institutional support) | Incremental movement toward dedicated spaces for breastfeeding counselling |
| Facilitators: peer collaboration, emotional engagement, policy alignment | Transformational Forums, PhotoVoice, advocacy, pledge, Breastfeeding Coordinator | Social (peer collaboration, networks, policy alignment); Motivated (emotional engagement) | Stronger provider collaboration; alignment with national priorities |
