# Supplementary material for: Can behavioral science advance breastfeeding-friendly primary care? Key findings from an evaluation in Kosovo
Source: PLOS Glob Public Health. 2025 Oct 31;5(10):e0005276. doi: 10.1371/journal.pgph.0005276 (PMC12578251; doi:10.1371/journal.pgph.0005276)
Supplement: S2 File — (DOCX) [file pgph.0005276.s002.docx]

**S2 File. Observation scoring guide**

**Clinical Skills: All Mothers**

| **Question** | **Description** |
| --- | --- |
| *a. Talk at all about infant feeding or how baby is being fed.* | Score YES if staff or mother talk at all about infant feeding or how baby is being fed. Note in the “General observations” field if it was the mother who raised the topic.  Score NO if staff or mother did not raise this topic. |
| *b. Give an explanation of follow up visits required* | Score YES if staff gave mother information if/when a follow up visit was required.  Score NO if staff or mother did not raise this topic. |
| *c. Request the Child Health Booklet* | Score YES if staff requested the Child Health Booklet to complete, or mother proactively provided it to staff.  Score NO if staff did not request or complete the Child Health Booklet.  Score N/A if the visit was not for routine infant care. |

**Clinical Skills: All Breastfeeding Mothers**

| **Question** | **Description -- adapted from (1-9)** |
| --- | --- |
| *d. Ask how breastfeeding was going* | Score YES if staff or mother talks at all about how breastfeeding is going.  Score NO if staff or mother did not raise this topic. |
| *e. Ask woman if she had any questions or concerns related to breastfeeding* | Score YES if staff asked mother if she had any questions or concerns about breastfeeding.  Score NO if staff or patient do not raise this topic. |
| *f. Answer questions or explain / demonstrate how to address concerns* | Score YES if staff correctly answered question/s or adequately explained/demonstrated how to address concern/s.  Score YES INC. if staff only partially or incorrectly answered question/s and inadequately explained/demonstrated how to address concern/s.  Score NO if staff did not answer question/s or explain how to address concern/s. |
| *g. Ask woman if people around her support her to breastfeed* | Score YES if staff asks directly or has any conversation about how the mother’s family or friends feels about breastfeeding and if she thinks they will be supportive.  Score NO if staff did not raise this topic. |
| *h. Discuss a woman's physiological ability to breastfeed* | Score YES if staff:   - Describe that nearly all women can breastfeed and that there are very few medical reasons *not* to breastfeed. Staff might also discuss some reasons why women might think they can’t breastfeed (mother’s illness/medications, smoking/alcohol, pregnancy, inverted nipples) and give her facts. Staff might ask if mother has heard any other reasons and dispel any myths.   Score YES INC. did discuss a woman's physiological ability to breastfeed but provided incorrect information.  Score NO if staff did not discuss a woman's physiological ability to breastfeed. |
| *i. Explain how to store and use expressed breastmilk* | Score YES if staff offered to, or did, explain how to store and use expressed breastmilk including reference to **both** of the following points:   - How to ensure safety and hygiene (washing hands, using clean equipment and suitable containers), storage times and methods (room temp, fridge, freezer). - Preparation: safe thawing/warming, milk separation (normal), using leftover milk.   Score YES INC. if staff if staff inadequately or incorrectly explained how to store and use expressed breastmilk.  Score NO if staff did not raise this topic. |
| *j. Talk about complementary feeding and benefits of continued breastfeeding* | Score YES if staff adequately cover **both** of the following points:   - Timely (start from 6 months age), adequate (amount, frequency and variety based on nutritional needs at each age), safe (preparation to avoid contaminants) and appropriate (correct consistency for age and given according to ‘responsive feeding’ principals). - Continued breastfeeding is important as “…it continues to provide up to half or more of a child’s nutritional needs during the second half of the first year, and up to one-third during the second year of life.”[45] Continued breastfeeding also provides a range of benefits beyond its nutritional value for babies (better protection from disease, SIDS, allergies, diabetes and obesity), for mother (better protection against breast and ovarian cancers and osteoporosis, helps with weight loss, lowers risk of PPD, saves time and money) and for both (increased bonding share, providing opportunities to share love, comfort and reassurance).   Score YES INC. if staff if staff inadequately or incorrectly discuss complementary feeding.  Score NO if no if staff or patient did not raise this topic. |
| *k. Give woman any information to take home about breastfeeding* | Score YES if staff gives mother take home information about breastfeeding.  Score NO if no take-home information is given. |
| *l. Tell woman about where to get information/support for breastfeeding* | Score YES if staff gave mother any information about where to get more advice or support for breastfeeding, either proactively or in response to a mother’s question.  Score NO if staff or patient did not raise this topic. |
| *m. Promote or provide samples of breastmilk substitutes* | Score YES if staff promoted or provided any breastmilk substitutes (formula). This does not include responding to a mother’s question about formula: staff should respond to any questions with factual information, however if when responding they appear to promote formula the score should be YES.  Score NO if staff did not raise this topic or responds to a mother’s question with only factual information and reaffirms that breastmilk is the ideal food for baby. |

**Clinical Skills: Breastfeeding Mothers with Babies Under Six Months**

| **Question** | **Description -- adapted from (1-9)** |
| --- | --- |
| *n. Ask mother if she is exclusively breastfeeding and explain benefits* | Score YES if staff explain along the following lines:   - Breastmilk is the ideal food for baby and provides 100% of its nutrition for the first six months. (“…and it continues to provide up to half or more of a child’s nutritional needs during the second half of the first year, and up to one-third during the second year of life.”[45]) - Supplementing with other liquids or foods will: 1) lessen mother’s milk supply; 2) baby will not get the same level of protective benefits; 3) reduce baby’s nutrient intake and for solids there is increased risk of choking, obesity and upset stomach.   Score YES INC. if staff inadequately explain importance or advise complementary feeding before six months.  Score NO if staff did not raise this topic. |
| *o. Explain importance of a good latch and different positions* | Score YES if staff describe **both** why a good latch is important and characteristics of a good latch along the following lines:   - Why a good latch is important: vital to successful breastfeeding, allows baby to effectively draw sufficient milk and mother to breastfeed without pain or injury. - Characteristics of a good latch: 1) Baby’s body is turned to mother and in a straight line, held close with head/neck/shoulders supported. 2) Baby’s mouth is wide open, lower lip not folded in, chin touching breast, more of areola below nipple in mouth. 3) Observe and/or hear baby making rhythmic burst-pause suckling and swallowing. - Positions: 1) Mother and baby should be well supported. 2) Baby-to-breast, not breast-to-baby. 3) Positions include cradle, cross-cradle, clutch (football), side-lie and laid-back. Each mother/baby dyad will have their own preference.   Score YES INC. if staff incorrectly describe why a good latch is important and/or characteristics of a good latch and/or positions.  Score NO if staff did not raise this topic. |
| *p. Observe mother breastfeeding* | Score YES if staff observed or asked to observe mother breastfeeding.  Score N/A if baby is not present with the mother during the visit.  Score NO if staff did not observe or ask to observe mother breastfeeding when there was a clear need to i.e. if mother was having problems breastfeeding. |
| *q. Explain responsive feeding* | Score YES if staff explain responsive feeding is the best way to feed baby and give a description along the following lines:   - Mother responds to baby’s cues and her own desire to nurse, and that breastfeeding is not just for nutrition but to share love, comfort and reassurance (see Unicef UK Infosheet for more information).   Score YES INC. if staff mention responsive feeding but describe it incorrectly or inadequately and revert to describing a ‘normal’ feeding pattern.  Score NO if staff did not raise this topic or instructs mother to feed baby on a schedule and for a certain time limit. |
| *r. Explain how to know if baby is getting enough milk* | Score YES if staff mention **all three** of the following:  1) Diaper output: in first 48 hours 2-3 wet diapers increasing to at least 6 wet diapers every 24 hours by day 5. Urine should be pale in color and not smelly. In first 48 hours babies pass a black tar-like stool (meconium) and this changes by day 4 to be green/yellow soft-to-runny stools. By day 4 babies should pass at least 2 stools, but the amount varies by baby and some pass a lot more and that is a good sign.  2) Baby is satisfied after feeding and finishes on its own. Mother’s breasts are likely to be softer.  3) Baby is healthy and gaining weight after two weeks of age (measured by a clinician).  Score YES INC. if staff mention less than 3 and/or is incorrect in the information they provide.  Score NO if staff did not raise this topic or mention any of the above points. |
| *s. Explain alternate ways of feeding baby e.g. cup, expression.* | Score YES if staff mentions **any** of the following **and** adequately describes how to do:   - Hand expression, cup feeding, spoon/syringe/dropper feeding, nursing supplementer device, finger feeding, bottle. - Staff should explain cup/spoon/syringe/dropper feeding must be done very carefully to avoid aspiration, and that mothers should be taught by a clinician.   Score YES INC. if staff mentions none of the above and/or did not adequately describe how to do them.  Score NO if staff did not raise this topic or mention any of the above points. |

**Clinical Skills: Non-Breastfeeding Mothers**

| **Question** | **Description** |
| --- | --- |
| *t. Ask mother if she had ever breastfed her baby.* | Score YES if staff or mother talk at all about breastfeeding.  Score NO if staff or mother did not raise this topic. |
| *u. Explain the value of breastfeeding and offer to help restart* | Score YES if staff explained the benefits of breastfeeding (for baby and mother) and offered help to restart. Staff should act in a respectful and considerate way.  Score YES INC. if staff inadequately explained benefits of breastfeeding, did not offer to help the mother restart, pressured the mother or made her feel bad for not breastfeeding.  Score NO if staff or mother did not raise this topic. |

**Interpersonal Skills**

The rating scale for each of the following skill components is as follows:

*1 = not at all. The staff do* ***none*** *of the behaviors in the “Description” column and this component needs a great deal of improvement.*

*2 = a little. The staff do* ***a small number*** *of the behaviors in the “Description” column and this component needs a lot of improvement.*

*3 = a moderate amount. The staff do* ***some*** *of the behaviors in the “Description” column and this component needs a moderate amount of improvement.*

*4 = a lot. The staff do* ***the majority*** *of the behaviors in the “Description” column and this component needs a little improvement.*

*5 = a great deal. The staff do* ***all*** *of the behaviors in the “Description” column and this component needs no improvement.*

| **Question** | **Description -- adapted from (3, 7)** |
| --- | --- |
| *a. Greets warmly and shows interest in woman* | - Greets the mother with customary welcomes e.g. "hello" "good morning" "how are you”. - Uses welcoming gestures e.g. standing up, shaking hands, gesturing for the mother to sit. - Greets other people accompanying the mother. |
| *b. Gives woman chance to ask questions/not hurried* | - Invites questions with a welcoming manner and responds positively. - Doesn’t check watch or usher mother out before she seems finished. |
| *c. Practices other supportive non-verbal communication* | - Uses tone, pace, eye contact, and posture that show care and concern. - Touches appropriately. - Sits at same level and near to mother and removes barriers e.g. desk, papers. - Avoids distractions e.g. checking phone, interruptions from others. |
| *d. Really listens to woman and understands concerns* | - Asks open questions: these usually start with "How? What? When? Where? Why?" e.g. "How is breastfeeding going for you?". - Shows interest e.g. uses gestures such as nodding and smiling, and simple responses e.g. "Mmm", or "Aha". - Reflects back what the mother says i.e. repeating back what a mother has said to show she has been heard, and to encourage her to say more e.g. if a mother says: "My baby was crying too much last night." The staff could say: "Your baby kept you awake crying all night?" |
| *e. Acts in a respectful and considerate way* | - Empathizes to show they understand mother’s feelings e.g. if a mother says: "My baby wants to feed very often and it makes me feel so tired," the staff could say something like: "You are feeling very tired all the time then?" not: "How often is he feeding? What else do you give him?". - Avoids words that sound judging e.g. right, wrong, well, badly, good, enough, properly. However, sometimes staff should use the "good" judging words to build a mother's confidence. |
| *f. Makes woman feel comfortable to express opinions/feelings/concerns* | - Accepts what a mother thinks and feels. - Staff responds to mother’s opinions, feelings and/or concerns in a neutral way, and does not agree or disagree. - Staff use skills such as reflecting back, showing interest and empathizing. |
| *g. Explains things well and gives practical help* | - Gives practical help e.g. tells mother things that she can do today, not in a few weeks. This might not be directly breastfeeding related but will support it e.g. when a mother feels tired give her ideas for getting more rest. - Gives a little relevant information e.g. staff gives only one or two pieces of information at a time. - Uses simple familiar terms to explain things to mothers and does not use technical medical terms. - Gives information in a positive way, so that it does not sound critical. |
| *h. Reassures woman and gives her confidence* | - Recognizes and acknowledges what a mother and baby are doing right e.g. A mother is breastfeeding her 3-month-old baby and giving drinks of fruit juice. The baby has slight diarrhea. The staff should respond by saying: “It is good that you are breastfeeding - breastmilk should help him to recover.” Not: “It is better not to give babies anything but breastmilk until they are about 6 months old.” |
| *i. Made suggestions that did not seem like orders* | - Instead of telling a mother what to do, the staff makes suggestions so the mother can decide which helps her feel in control and more confident. - Suggestions can be in the form of a statement, a question or a question followed by a statement e.g. instead of saying: “Keep the baby in bed with you so that he can feed at night!” staff could say: “It might be easier to feed him at night if he slept in bed with you.” Or “Would it be easier to feed him at night if he slept with you?” Or “How would you feel about letting him sleep in bed with you? It might be easier to feed him that way.” |

**General Observations**

In the General Observations field:

- Note if the mother (or anyone accompanying her) was the first one to raise the issue of infant feeding.
- Describe any visual aids that were used in answering questions, describing aspects of breastfeeding or overcoming problems.
- Briefly note the power relationships and dynamic between staff and mother (and anyone with patient e.g. husband, grandmother). Did the staff play out their role as expected i.e. caring medical provider, authority position? Did the staff express an obvious attitude (positive or negative) towards the mother in general or the topic of breastfeeding, and if so describe?

**References**

1. World Health Organization, UNICEF. Appendix: Indicators for monitoring: protecting, promoting and supporting Breastfeeding in facilities providing maternity and newborn services: the revised Baby-Friendly Hospital Initiative. Geneva, Switzerland2018.

2. World Health Organization, UNICEF. Implementation guidance: protecting, promoting and supporting breastfeeding in facilities providing maternity and newborn services – the revised Baby-friendly Hospital Initiative. Geneva, Switzerland2018.

3. World Health Organization. Counselling for Maternal and Newborn Health Care: A Handbook for Building Skills. 2013.

4. Unicef UK. Unicef UK Baby Friendly Initiative Infosheet: Responsive Feeding. 2016.

5. World Health Organization. Infant and Young Child Feeding: Model Chapter for Textbooks for Medical Students and Allied Health Professionals. ANNEX 1, Acceptable medical reasons for use of breast-milk substitutes. Geneva; 2009.

6. World Health Organization, UNICEF. Breastfeeding Counselling a Training Course. Geneva; 1993.

7. World Health Organization, UNICEF. Caring for newborns and children in the community: a training course for community health workers. Geneva; 2015.

8. World Health Organization. Guideline: counselling of women to improve breastfeeding practices. Geneva; 2018.

9. World Health Organization. WHO recommendations on postnatal care of the mother and newborn. Geneva; 2013. Report No.: ISBN 978 92 4 150664 9.
