# Supplementary material for: Can behavioral science advance breastfeeding-friendly primary care? Key findings from an evaluation in Kosovo
Source: PLOS Glob Public Health. 2025 Oct 31;5(10):e0005276. doi: 10.1371/journal.pgph.0005276 (PMC12578251; doi:10.1371/journal.pgph.0005276)
Supplement: S3 File — (DOCX) [file pgph.0005276.s003.docx]

**S3 File Observation checklist – English**

**Observation Tool: After Birth Consultations (AB)**

Q.1 Date: ______   Q.2 FMC number: ______   Q.3 Patient number: ______   Q.4 Staff number: ______

Q.5 Staff position: __Dr __Nurse __Midwife     Q.6 Staff sex: __F __M   Q.7 Observer name: _________________

Q.8 Obs. duration: ____ minutes (start: ______ end: ______ )   Q.9 Baby’s age: ____ (months) __don’t know

Q.10 Is mother currently breastfeeding: __yes  __no  __don’t know

Q.11 Visit type: __postnatal check for mother   __postnatal check for baby   __immunization visit for baby

__routine check for baby   __acute visit for baby   __acute visit for mother   __other

Q.12 Other staff present: __yes (__Dr __Nurse __Midwife)  __no

Q.13 Was anyone with the mother: __yes (__Husband __Child __Grandmother __Other)  __no

-------------------------------------------------------------------------------------------------------------------------------

AB.1 Clinical Skills *(1 = yes, 2 = yes but gave incorrect/incomplete information, 3 = no, 4 = N/A)*

*For breastfeeding mothers of babies <6m check questions a-s; For breastfeeding mothers of babies >6m check questions a-m for non-breastfeeding mothers check a-c+t-u:*

***All mothers:***

1. Talk about infant feeding or breastfeeding or how baby is being fed? 1    2    3    4
2. Give her an explanation of follow up visits required 1    2    3    4
3. Request the Child Health Booklet *(only for routine infant care visits)* 1    2    3    4

***All breastfeeding mothers:***

1. Ask how breastfeeding was going? 1    2    3    4
2. Ask if mother had any questions or concerns related to breastfeeding 1    2    3    4
3. Answer questions or explain how to address concerns 1    2    3    4
4. Ask mother if people around her support her to breastfeed 1    2    3    4
5. Discuss a woman's physiological ability to breastfeed 1    2    3    4
6. Explain how to store and use expressed breastmilk 1    2    3    4
7. Talk about complementary feeding and benefits of continued breastfeeding 1    2    3    4
8. Give mother any information to take home about breastfeeding 1    2    3    4
9. Tell mother about where to get information/support for breastfeeding 1    2    3    4
10. Promote or provide samples of breastmilk substitutes 1    2    3    4

***Breastfeeding mothers with babies under six months***

1. Ask mother if she is exclusively breastfeeding and explain benefits 1    2    3    4
2. Explain importance of a good latch and show different breastfeeding positions 1    2    3    4
3. Observe mother breastfeeding 1    2    3    4
4. Explain responsive feeding (feeding cues, unrestricted frequency/length) 1    2    3    4
5. Explain how to know if baby is getting enough milk 1    2    3    4
6. Explain alternate ways of feeding baby e.g. cup, expression 1    2    3    4

***Non-Breastfeeding mothers***

1. Ask mother if she had ever breastfed her baby 1    2    3    4
2. Explain the value of breastfeeding and offer to help restart 1    2    3    4

AB.2 Interpersonal Skills *(1 = not at all, 2 = a little, 3 = a moderate amount, 4 = a lot, 5 = a great deal)*

1. Greet the mother warmly and show interest in her and her baby 1   2   3   4   5
2. Give the mother the chance to ask questions and didn’t seem hurried 1   2   3   4   5
3. Practice other supportive non-verbal communication* 1   2   3   4   5
4. Really listen to the mother and understand her concerns 1   2   3   4   5
5. Act in a respectful and considerate way 1   2   3   4   5
6. Make the mother feel comfortable to express opinions/feelings/concerns 1   2   3   4   5
7. Explain things well and give practical help in a way she could understand 1   2   3   4   5
8. Reassure the mother and give her confidence 1   2   3   4   5
9. Make suggestions that did not seem like orders 1   2   3   4   5

**Will not be validated with patient exit interview*

AB.3 Change in engagement when infant feeding discussed

*(1 = more engaged, 2 = less engaged, 3 = no change, 4 = N/A as infant feeding not discussed)*

1. Staff 1    2    3    4 b.   Woman 1    2    3    4

AB.4 General observations

_______________________________________________________________________________

_______________________________________________________________________________

_______________________________________________________________________________

_______________________________________________________________________________

_______________________________________________________________________________

**Observation checklist – Albanian**

**Mjeti i observimit: Konsultimet pas lindjes (AB)**

Q.1 Data: ______ Q.2 Numri i QMF-së: ______ Q.3 Numri i pacientit: ______ Q.4 Numri i stafit: ______

Q.5 Pozita e stafit: __Dr __Infermiere __Mami Q.6 Gjinia e stafit: __F __M Q.7 Emri i observuesit: _______________

Q.8 Kohëzgjatja e observimit: ____ minutes (filloi: ______ mbaroi: ______ ) Q.9 Mosha e foshnjës/fëmiut: ____ (muaj) __nuk e di

Q.10 A është nëna momentalisht duke ushqyer fëmiun me gji: __po __jo __nuk e di

Q.11 Lloji i vizitës: __ kontroll pas lindjës-nëna __ kontroll pas lindjës-fëmiu __ vizitë për vakcinim të fëmiut

__ vizitë rutinore-fëmiu __ kujdes akut-fëmiu __ kujdes akut-nëna __tjetër

Q.12 Kishte staf tjetër që ishte prezent: __po (__Dr __Infermiere __Mami) __jo

Q.13 A ishte nëna e shoqëruar nga dikush: __po (__burri __fëmiju__gjyshja __tjetër) __jo

-------------------------------------------------------------------------------------------------------------------------------------------

AB.1 Aftësitë klinike *(1 = po, 2 = po por ishin gabim/informata gjysmake, 3 = jo, 4 = Nuk është pergjigjur)*

*Për nënat që ushqejnë fëmiun me gji <6m shiqoni pyetjet a-s; Për nënat që ushqejnë fëmiun me gji >6m shiqoni pyetjet a-m për nënat që nuk ushqejnë fëmiun me gji shiqoni pyetjet a-c+t-u:*

***Për të gjitha nënat:***

1. I ka treguar për ushqyerjen e foshnjës ose gjidhënien ose ka biseduar 1 2 3 4

për mënyrën e ushqyerjes së fëmiut të gruas

1. I ka shpjeguar për vizitën e rradhës që duheni t’a bëjë 1 2 3 4
2. Ka kërkuar fletoren/kartelen shëndetësore të fëmiut *(vetëm për vizitat e foshnjeve).* 1 2 3 4

***Për të gjitha nënat që ushqejnë fëmiun me gji:***

1. I ka pyetur se si është duke shkuar gjidhënia 1 2 3 4
2. I ka pyetur se a kanë ndonjë pyetje apo shqetësim rreth gjidhënies 1 2 3 4
3. I është përgjigjur pyetjeve ose ju ka shpjeguar se si t’i adresojnë shqetësimet 1 2 3 4
4. E ka pyetur nënën se a e mbështesin në të ushqyerit me gji personat që 1 2 3 4

ka në rrethin e saj

1. Diksutoi për aftësinë fizilogjike të gruas për të ushyqer me gji 1 2 3 4
2. E ka bërë nënën të ndihet se mund të ushqenjë me gji fëmiun e sja në 1 2 3 4

mënyrë të susksesshme

1. I ka shpjeguar mënyrën e ruajtjes dhe përdorimit të qumështit të gjirit 1 2 3 4
2. Ka biseduar për ushqimin plotësues si dhe për përfitimet e ushqyerjes të 1 2 3 4

vazhdueshme me gji

1. I ka dhënë nënës ndonjë infromacion (broshurë) në lidhje me ushqyerjen 1 2 3 4

me gji që ajo ta merr në shtëpi

1. I ka treguar nënës se ku mund te gjejë infromata/mbeshtetje rreth gjidhenies 1 2 3 4
2. E ka nxitur ose I ka ofruar mostra për të përdorur si zëvendësim të 1 2 3 4

qumështit të gjirit (psh. qumësht formule) 1 2 3 4

***Vetëm për nënat që ushqejnë me gji fëmiun nën gjashtë muaj***

1. E ka pyetur nënën se a është duke e ushqyer femiun eksluzivisht me gji 1 2 3 4

dhe i ka shpjeguar benefitet

1. I ka shpejguar rëndësinë e puthitjes të mirë dhe i ka treguar pozicione 1 2 3 4

të ndryshme që mund të merr gjatë gjidhënies

1. E ka shiquar se si jep gji 1 2 3 4
2. I ka shpjguar të ushqyerit ushqyerjen sipas kërkeses (mëkimet, frekuenca/ 1 2 3 4

koha e zjgatjes e pakufizuar)

1. I ka shpjeguar se si mund t’a dijë nëse fëmiu i saj ëashtë duke thithur 1 2 3 4

sasi të mjaftueshme të qumështit

1. I ka shpjeguar mënyra alternative për t’a ushqyer fëmiun e saj psh me 1 2 3 4

filxhan, pompë

***Për nënat që nuk ushqejnë fëmiun me gji***

1. E ka pyetur se a e ka ushqyer fëmiun e saj ndonjëherë me gji 1 2 3 4
2. I ka shpejguar për rëndësinë e të ushqyerit me gji dhe i ka ofruar t’i 1 2 3 4

ndihmojë të fillojnë

AB.2 Aftësitë ndërpersonale *(1 = aspak, 2 = pak, 3 = konsiderueshëm, 4 = shumë, 5 = tepër shumë)*

1. Ka përshëndetur guran ngrohtësisht dhe ka treguar interesim për të dhe 1 2 3 4 5

fëmiun e saj

1. I dha mundësinë gruas t’i bënte pyetje dhe nuk dukej se nxitonte 1 2 3 4 5
2. Praktikoi forma të tjera të mbështetjes, jo verbale* 1 2 3 4 5
3. Me të vërtetë dëgjoi gruan dhe kuptonte shqetësimet e saj 1 2 3 4 5
4. Është sjellur me respekt dhe konsideratë 1 2 3 4 5
5. E ka bërë gruan të ndihet rehat për të treguar lirshëm mendimet, ndjenjat 1 2 3 4 5

dhe shqetësimet e saj

1. Ju shpjegoi gjërat mirë dhe ju dha ndihmë praktike që ajo t’a kuptonte më mire 1 2 3 4 5
2. E siguroi gruan për veprimet dhe i dha vetëbesim 1 2 3 4 5
3. I dha sugjerime që nuk tingëllonin si urdhëra 1 2 3 4 5

**Nuk do të vërtetohet me intervistën në dalje të pacientit*

AB.3 Ndryshimi në përfshirjen kur të ushqyerit e foshnjës/fëmiut u diskutuan

*(1 = përfshirje të madhe, 2 = përfshirje të vogël, 3 = nuk kishte ndryshim, 4 = nuk kishte përgjigje pasi që nuk u diskutua rreth mënyrës së ushqimit të fëmiut)*

1. Stafi 1 2 3 4 b. Gruaja 1 2 3 4

AB.4 Observimet gjenerale

_______________________________________________________________________________
