# Supplementary material for: Can behavioral science advance breastfeeding-friendly primary care? Key findings from an evaluation in Kosovo
Source: PLOS Glob Public Health. 2025 Oct 31;5(10):e0005276. doi: 10.1371/journal.pgph.0005276 (PMC12578251; doi:10.1371/journal.pgph.0005276)
Supplement: S4 File — (DOCX) [file pgph.0005276.s004.docx]

**S4 File. Client exit interview questionnaire – English**

**Exit Interview Tool: After Birth Patients (AB)**

*DO NOT ASK MOTHER THESE QUESTIONS:*

Q.1 Date: ______   Q.2 FMC number: ______   Q.3 Patient number: ______   Q.4 Staff number: ______

Q.5 Interviewer name: __________   Q.6 Interview duration: ____ minutes (start: ________ end: ______ )

Q.7 Others present: __yes (who: ________________)  __no   Q.8 Baby’s age: __ months

Q.9 Visit type: __ postnatal check mother __ postnatal check baby __ immunization visit baby __ routine check baby __ acute care baby __ acute care mother __other

--------------------------------------------------------------------------------------------------------------------------------

***START OF INTERVIEW***

AB.1 What is your age?

____(years)

AB.2 What is the highest level of school you have attended?

__pre-primary   __primary   __lower secondary   __upper secondary   ___higher

AB.3 To what ethnic group do you belong?

__Albanian   __Serbian   __Other   __Did not answer

AB.4 Do you have any other children?

__ yes  __no AN.4.1 If yes, were they ever breastfed? __ yes   __ no

AB.5 What is the most important thing you learned today during your consultation?

______________________________________________________________________________

AB.6 How would you rate your overall experience at the FMC today? *(show response card #1)*

*1 = very bad, 2 = bad, 3 = average, 4 = good, 5 = very good, 6 = don’t know/didn’t answer*

1    2    3    4    5    6

AB.7 Before today, did a health care provider or community worker talk with you about breastfeeding:

*1= yes, 2=no, 3=don’t know/didn’t answer/not applicable*

1. During your pregnancy 1    2    3
2. During the first two days after baby’s birth 1    2    3
3. Anytime within the first month of baby’s birth 1    2    3

AB.8 Do you currently breastfeed your baby?

__ yes exclusive   __ yes mixed (BM + formula/other)   __ no but previously did   __ no never

AB.9 Do you believe that you can successfully breastfeed your baby? *(show response card #2)*

*1 = not at all, 2 = a little, 3 = a moderate amount, 4 = a lot, 5 = a great deal, 6 = don’t know/not applicable*

1     2    3    4    5    6

AB.10 After your consultation today have your feelings changed about: *(show response card #2)*

*1 = not at all, 2 = a little, 3 = a moderate amount, 4 = a lot, 5 = a great deal, 6 = don’t know or N/A*

1. The value of breastfeeding for your baby’s health 1    2    3    4    5    6
2. The length of time you will breastfeed your baby 1    2    3    4    5    6
3. Your ability to overcome breastfeeding challenges (current or future) 1    2    3    4    5    6

AB.11 During your consultation today did the healthcare provider:

*1= yes, 2=no, 3=don’t know, 4=N/A*

1. Talk about infant feeding or breastfeeding or how your baby is being fed? 1    2    3    4
2. Explain the benefits of breastfeeding (exclusive BF under six months or continued BF until baby is 2+ years) 1    2    3    4
3. Explain that most women are able to breastfeed (physiological ability) 1    2    3    4
4. Ask if you had any questions or concerns related to breastfeeding 1    2    3    4
5. Tell you about where to get information/support for breastfeeding 1    2    3    4
6. Give you any information to take home about breastfeeding 1    2    3    4
7. Ask if people around you support you to breastfeed 1    2    3    4
8. Promote or provide samples of breastmilk substitutes 1    2    3    4
9. Give you an explanation of follow up visits required 1    2    3    4
10. Observe you breastfeeding 1    2    3    4

AB.12 During your consultation today do you feel like the healthcare provider: *(show response card #2)*

*1 = not at all, 2 = a little, 3 = a moderate amount, 4 = a lot, 5 = a great deal, 6 = didn’t answer/don’t know*

1. Really listened to you and understood your concerns 1   2   3   4   5   6
2. Made you feel comfortable to express your opinions, feelings and concerns 1   2   3   4   5   6
3. Explained things well and gave practical help in a way you could understand 1   2   3   4   5   6

AB.13 Who is the first person you would turn to for questions or problems about feeding your baby? Who would you turn to next? *(show response card #3)*

     Husband Mother/Mother-in-Law Friend Religious leader     Doctor Nurse/Midwife Internet

AB.14 Can you tell me if you agree or disagree with the following statements:

*1 = agree, 2 = disagree, 3 = don’t know/didn’t answer*

1. A mother should start breastfeeding during the first hour after birth 1    2    3
2. A baby should be fed only breastmilk for the first six months 1    2    3
3. The health benefits of breastfeeding are minimal after baby turns one year 1    2    3
4. Breastfeeding is good for the mother’s health 1    2    3
5. Formula is just as nutritious for babies as breastmilk 1    2    3
6. A baby needs to drink water in addition to breastmilk 1    2    3
7. If a mother breastfeeds, the baby will have fewer illnesses, like diarrhea 1    2    3
8. Formula feeding is more convenient than breastfeeding 1    2    3

AB.15 What kind of support would be most helpful for you to successfully breastfeed?

*(If mother is having difficulty answering prompt with ALL of the following examples: classes at the FMC, help at home, mother support groups, a free phone number to call with questions, a website or APP, pamphlets with information, more information during prenatal check-ups. Record if a prompt was made by noting “Prompted”)*

______________________________________________________________________________

--------------------------------------------------------------------------------------------------------------------------------

***END OF INTERVIEW***

AB.16 Other comments/observations: ____________________________________________________

______________________________________________________________________________

**Client exit interview questionnaire – Albanian**

**Mjeti i intervistimi në dalje: Pacientët pas lindjes**

*MOS I PYESNI NËNËS KËTO PYETJE:*

Q.1 Data e intervistës: ______   Q.2 Numri i QMF-së:______   Q.3 Numri i pacientit: ______

Q.4 Numri i stafit: ______   Q.5 Emri i intervistuesit: _______________

Q.6 Kohëzgjatja e intervistës: ____ minuta (filloi: ______ mbaroi: ______ )

Q.7 Persona të tjerë prezent: __po (kush: _______________________)  __jo  Q.8 Mosha e fëmiut: __ muaj

Q.9 Lloji i vizitës: __ kontroll pas lindjës-nëna   __ kontroll pas lindjës-fëmiu     __ vizitë për vakcinim të fëmiut

   __ vizitë rutinore-fëmiu/foshnja     __ kujdes akut-fëmiu     __ kujdes akut-nëna     __tjetër

--------------------------------------------------------------------------------------------------------------------------------

***FILLIMI I INTERVISTËS***

AB.1 Sa vjec jeni? ____(vitet)

AB.2 Cili është niveli më i lartë i shkollimit që keni arritur?

__para-shkollor   __fillore   __i mesëm i ulët   __i mesëm i lartë   __i lartë

AB.3 Cilit grup etnik i përkisni? __Shqiptar  __Serb  __Tjetër __Nuk është përgjigjur

AB.4 A keni ndonjë fëmijë tjetër?

__po __jo AB.4.1 Nëse po, a i keni ushqyer ata me gji? __ po __ jo

AB.5 Cila është gjëja më e rëndësishme që keni mësuar sot gjatë konsultimit tuaj?

______________________________________________________________________________

AB.6 Si do ta vlerësonit eksperiencën tuaj në QMF sot? *(tregoni kartën e përgjigjes #1)*

*1 = shumë keq, 2 = keq, 3 = mesatare, 4 = mirë, 5 = shumë mirë, 6 = nuk e di/nuk është përgjigjur*

1    2    3    4    5    6

AB.7 Para konsultimit tuaj sot, a ka biseduar me ju rreth gjidhënies ndonjë ofrues i shërbimit shëndetësorë apo punëtorë të komuniteti: *1= po, 2=jo, 3= nuk e di/nuk është përgjigjur/nuk aplikohet*

1. Gjatë shtatëzanisë suaj 1    2    3
2. Gjatë dy ditëve të para paslindjes 1    2    3
3. Kur do gjatë muajit të parë pas lindjes 1    2    3

AB.8 A e ushqeni me gji momnetalisht fëmiun tuaj?

__ po ekskluzivisht   __ po ushqim të përzier (QGJ + formula/tjetër)

__ jo por më herët e kam ushqyer   __ jo kurr

AB.9 A besoni se mund ta ushqeni me gji femiun tuaj në mënyrë të suksesshme? *(tregoni kartën e përgjigjeve #2)*

1    2    3    4    5    6

AB.10 Pas konsultimit të sotëm a keni ndryshuar mendimin tuaj për: *(tregoni kartën e përgjigjeve #2)*

*1 = aspak, 2 = pak, 3 = konsiderueshëm, 4 = shumë, 5 = tepër shumë, 6 = nuk e di/nuk është përgjigjur*

1. Rëndësinë që ka të ushqyerit me gji për shëndetin e fëmiut tuaj   1    2    3    4    5    6
2. Kohezgjatjen që duhet ta ushqeni fëmiun tuaj                 1    2    3    4    5    6
3. Aftësinë tuaj për të tejkaluar sfidat e gjidhënies (tani ose në të ardhmen)        1    2    3    4    5    6

AB.11 Gjatë konsultimit tuaj sot ofruesi i sherbimit shëndetësorë: *1= po, 2=jo, 3=nuk e di, 4=nuk është përgjigjur*

1. Ju ka treguar për ushqyerjen e foshnjes apo gjidhëninen apo se si është     1    2    3    4

duke u ushqyer fëmiu juaj?

1. Ju ka treguar benefitet e gjidhenies (per gjidhenien ekskluzive nën gjashtë muaj ose vazhdimin e saj deri sa femiu mbush 2+ vjet) 1    2    3    4
2. Ju ka shpjeguar se shumica e grave janë në gjendje të ushqejne me gji femiun e tyre

(aftësinë fiziologjike te gruas) 1    2    3    4

1. Ju ka pyetur se a keni ndonjë pyetje apo shqetësim rreth gjidhënies         1    2    3    4
2. Ju ka treguar se ku mund te gjeni infromata/mbeshtetje rreth gjidhenies   1    2    3    4
3. Ju ka dhënë ndonjë infromacion (broshurë) në lidhje me ushqyerjen me   1    2    3    4

gji që ju ta merrni në shtëpi

1. Ju ka pyetur se a ju mbeshtesin në të ushqyerit me gji personat që keni në rrethin tuaj 1    2    3    4
2. Ju ka nxitur ose ju ka ofruar mostra për të përdorur qumësht formule   1    2    3    4
3. Ju ka shpjeguar për vizitën e rradhës që duheni t’a bëni           1    2    3    4
4. Ju ka shiquar se si jepni gji   1    2    3    4

AB.12 Gjatë konsultimit tuaj sot mendoni se ofruesi i shërbimit shëndetësor: *(tregoni kartën e përgjigjeve #2)*

*1= aspak, 2 = pak, 3 = konsiderueshëm, 4 = shumë, 5 = tepër shumë, 6 = nuk e di/nuk është përgjigjur*

1. Me të vërtetë ju dëgjoi dhe kuptonte shqetësimet e juaja             1   2   3   4   5   6
2. Ju ka bërë të ndjeheni rehat për të treguar lirshëm mendimet, ndjenjat                1   2   3   4   5   6

dhe shqetësimet tuaja

1. Ju shpjegoi gjërat mirë dhe ju dha ndihmë praktike që ju t’a kuptoni më mire      1   2   3   4   5   6

AB.13 Kush është personi i parë që do t’i drejtoheshit për pyetje ose në rast të ndonjë problemi me të ushqyerit e fëmiut tuaj? Kujt do t’i drejtoheshit më pas? *(tregoni kartën e përgjigjeve #3)*

Bashkëshortit Nënës/Vjehrrës Shoqes/Shokut Internet

Udhëheqësit fetar (psh. Hoxhës, Priftit etj) Mjekut Infermieres/Mamisë

AB.14 A mund të më tregoni nese jeni dakrod apo nuk pajtoheni me deklaratat e mëposhtme:

*1 = pajtohem, 2 = nuk pajtohem, 3 = nuk e di/nuk është pergjigjur*

1. Nëna duhet të filloj me gjidhënie gjatë orës se parë pas lindjes 1    2    3
2. Foshnja duhet të ushqethet vetëm me gji për gjashtë muajt e pare 1    2    3
3. Përfitimit shëndetësore të ushqyerjes me gji janë minimale pasi që fëmiu mbush një vit 1    2    3
4. Ushqyerja me gji është e mirë për shëndetin e nënës 1    2    3
5. Formula ka vlera ushqyese të barabarta me qumështin e gjirit 1    2    3
6. Foshnjet përvec qumështit të gjirit duhet të pijnë edhe ujë 1    2    3
7. Nëse nëna e ushqen fëmiun me gji, fëmiu do të ketë më pak sëmundje si barkëqtija 1    2    3
8. Të ushqyerit me anë të formulës është më i përshtatshëm se sa të ushqyerit me gji 1    2    3

AB.15 Cfarë lloj ndihme do të ishte më e dobishme për ju në mënyrë që të keni sukses në gjidhënie?

*(Nëse nëna ka vështirrësi për tu përgjijgur përmendni TË GJITHË shembujt: klasa në QMF, ndihmë në shtëpi, grupe për mbështjetjen e nënave, një numër pa pagesë për t’a thirrur në rast të pyetjeve, një ëeb-faqe ose aplikacion, broshurë me infromata, më shumë informata gjatë kontrollave para lindjes (pre-natale). Shënoni “Tregova shembuj” nëse ju keni ndërhyrë duke i treguar shembuj.*

____________________________________________________________________________________

***FUNDI I INTERVISTËS***

AB.16 Komente të tjera/observimet: ____________________________________________________

**Interview Response card #1 / KARTA PËR PËRGJIGJET E INTERVISTES #1**


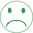

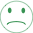

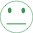

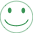

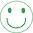


1 2 3 4 5

Shumë keq Keq Mesatar/Mesatarisht Mirë Shumë mirë

**Interview Response Card #2 / KARTA PËR PËRGJIGJET E INTERVISTES #2**

_____
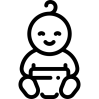

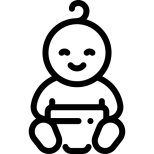

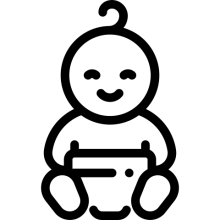

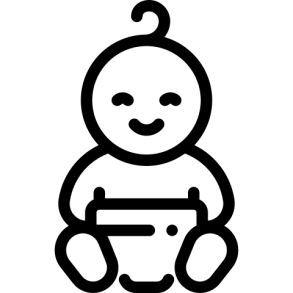


1 2 3 4 5

Aspak Pak Konsiderueshëm Shumë Tepër shumë

**Interview Response Card #3 / KARTA PËR PËRGJIGJET E INTERVISTES #3**

**Bashkëshorti Nëna / vjehrra Miqtë/Shoqet Internet**


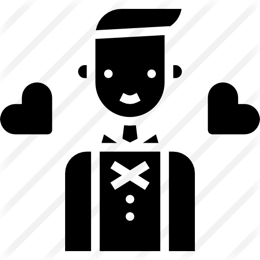

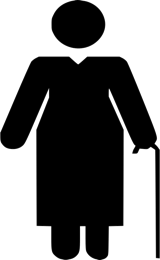

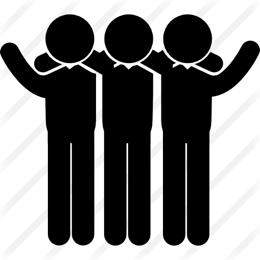

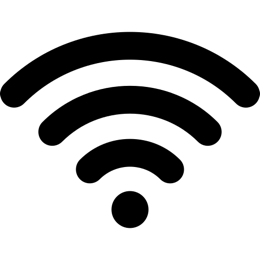


**Udhëheqësit fetarë Mjeku Infermierja / Mamia**


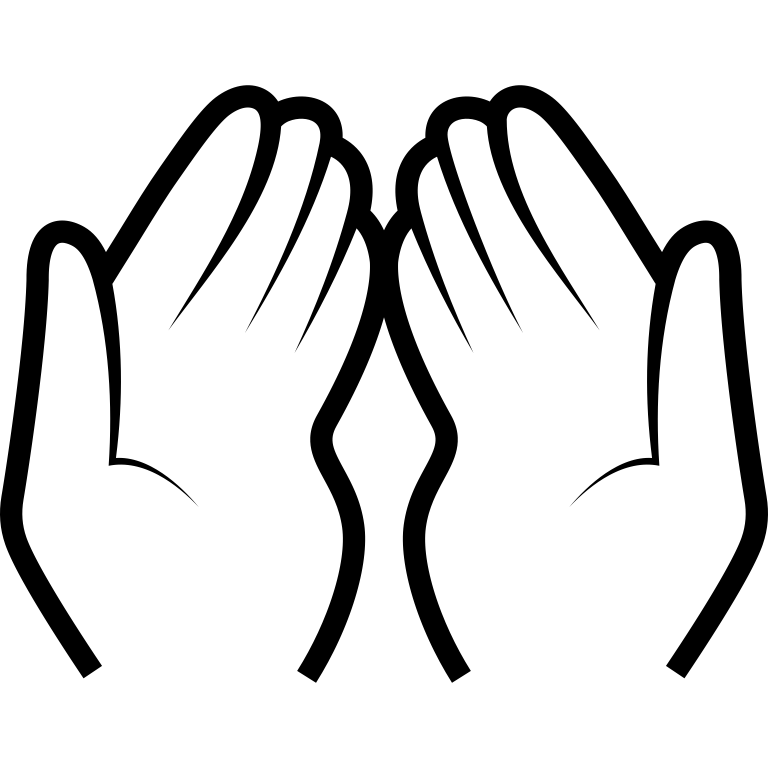

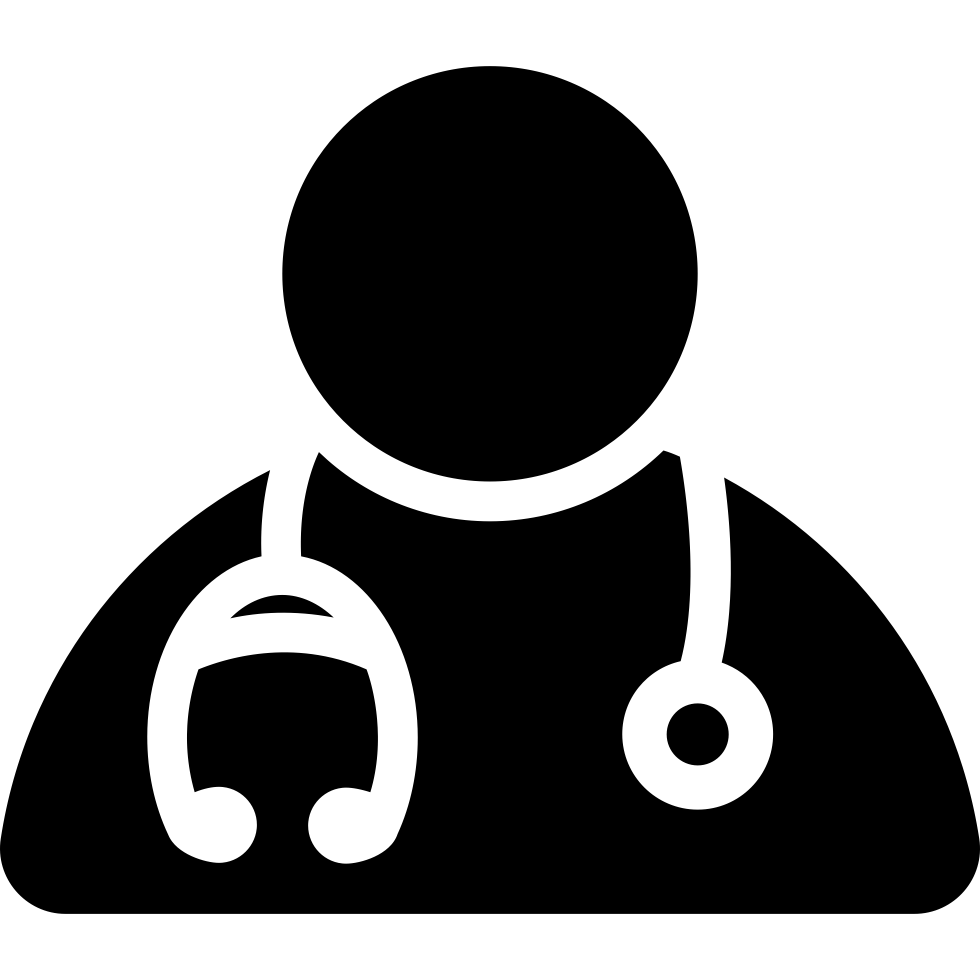

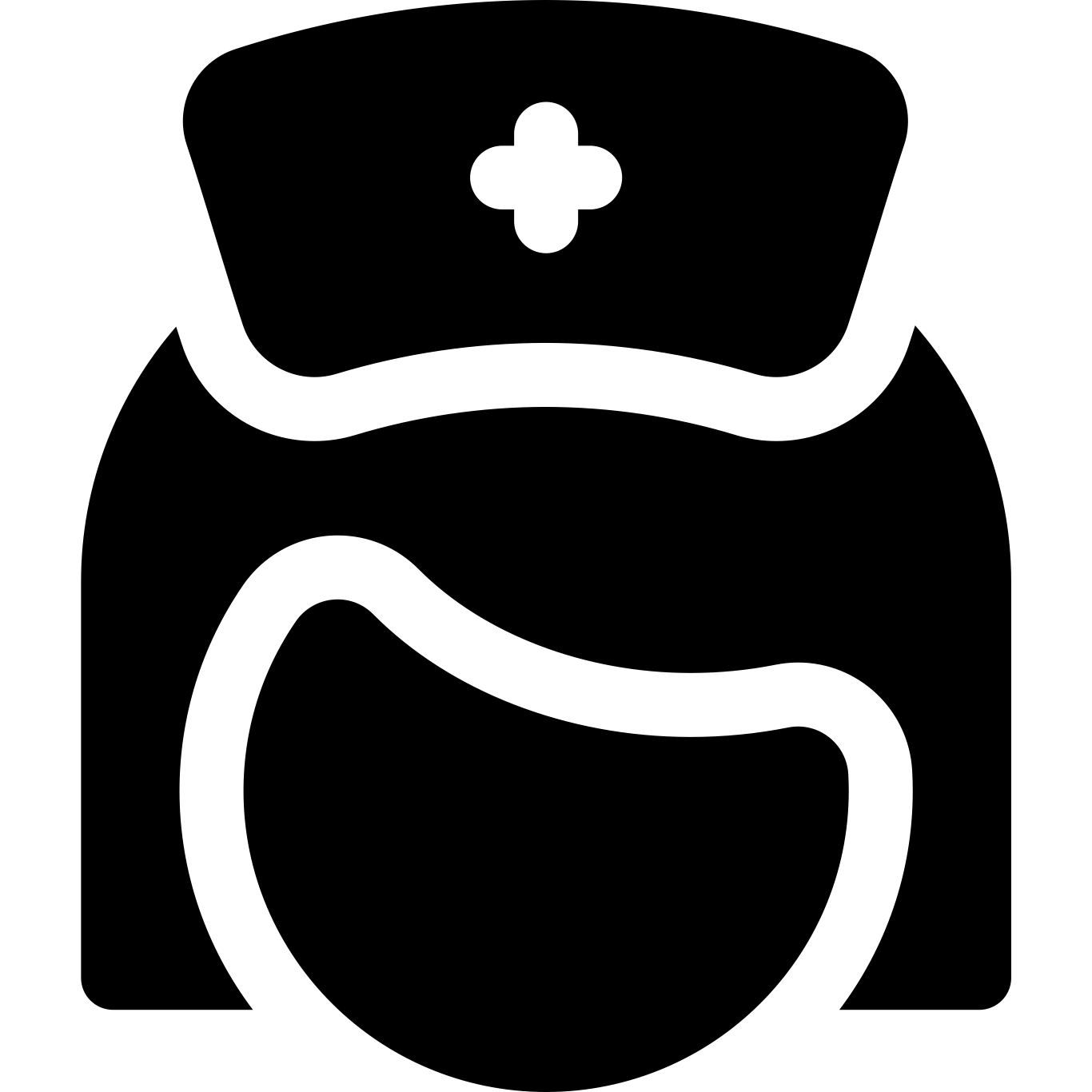


*Icons made by Freepik from www.flaticon.com*
