# Supplementary material for: Can behavioral science advance breastfeeding-friendly primary care? Key findings from an evaluation in Kosovo
Source: PLOS Glob Public Health. 2025 Oct 31;5(10):e0005276. doi: 10.1371/journal.pgph.0005276 (PMC12578251; doi:10.1371/journal.pgph.0005276)
Supplement: S5 File — (DOCX) [file pgph.0005276.s005.docx]

**S5 File. Provider interview questionnaire**

## **Structured Interview Tool: Clinical Family Medicine Center (FMC) Staff**

Q.1 Date: ______ Q.2 FMC number: ______ Q.3 Staff number: ______

Q.4 Staff sex: __F __M Q.5 Staff position: __Dr __Nurse __Midwife

Q.6 Interviewer name: ______________Q.7 Interview duration: ____ minutes (start: ______ end: ______ ) Q.8 Others present: __yes (who: ________________) __no

------------------------------------------------------------------------------------------------------------------------------------

D.1 In what year were you born? _______

D.2 To what ethnic group do you belong?

__Albanian __Serbian __Other __Did not answer

D.3 What year did you graduate from medical school? ______________

D.4 When did you join this FMC? *(month/year)?* ______________

D.5 Have you received any training in breastfeeding and lactation management?

a. In medical school: __ yes __ no __ don’t know

b. Since medical school: __ yes (year: __ # hours: __) __ no __ don’t know

c. On the job at this FMC: __ yes (year: __ # hours: __) __ no __ don’t know

## **INTERVENTION *(these questions were only asked at endline)***

I.1 a. I am going to read a list of project activities and ask you whether you recall each one. *(interviewer: record yes or no for each item listed*)

b For each project activity that you recall, I will ask for your opinion on how likely the activity is to improve the protection, promotion and support of breastfeeding at your FMC: not at all, a little or a lot? (*Interviewer: for each project activity listed circle 1 for not at all, 2 for a little, 3 for a lot or 4 for not recalled by respondent)*

1. Breastfeeding Forum in Pristina (December 2019) __ yes __ no 1 2 3 4
2. Video documenting the Breastfeeding Forum __ yes __ no 1 2 3 4

(shared by Viber and on Facebook in early 2020)

1. Breastfeeding training via Zoom (in April 2021 __ yes __ no 1 2 3 4
2. Video on breastfeeding support in antenatal care __ yes __ no 1 2 3 4
3. Video on breastfeeding support in infant care __ yes __ no 1 2 3 4
4. Video on breastfeeding support during vaccinations __ yes __ no 1 2 3 4
5. Breastfeeding posters at your FMC __ yes __ no 1 2 3 4
6. FMC Breastfeeding Pledge __ yes __ no 1 2 3 4
7. Facebook stories about breastfeeding support at __ yes __ no 1 2 3 4

your FMC or another FMC

1. Breastfeeding brochures for families __ yes __ no 1 2 3 4
2. Clinician Breastfeeding APP __ yes __ no 1 2 3 4
3. Breastfeeding Coordinators elected for your FMC __ yes __ no 1 2 3 4
4. You and your colleagues taking photos to depict __ yes __ no 1 2 3 4

your work helping mothers to breastfeed (in 2020)

I.2 From the list of project activities I just read, chose the intervention activity you think has the highest likelihood of improving the protection, promotion and support of breastfeeding at your FMC and explain why you think it can be so helpful.

1. Intervention activity: _________________________________________________
2. Why helpful:________________________________________________________

I.3 On a scale of 1 to 3 (1=Not at all, 2=A little, 3=A lot), do you think this project:

1. Changed the way you and your colleagues value breastfeeding at this FMC? 1 2 3
2. Improved understanding of how to make practices more breastfeeding friendly

at your FMC?

1 2 3

1. Has resulted in more protection, promotion and support of breastfeeding

at your FMC?

1 2 3

1. Will in the future result in more protection, promotion and support of breastfeeding

at your FMC?

1 2 3

I.4 Please provide some description for your answer to I.3d i.e. why or why not will this project make your FMC more breastfeeding friendly?

___________________________________________________________________

I.4 Should the following activities be scaled to other FMCs in Kosovo?

1. Clinician APP __ yes __ no
2. Videos on breastfeeding support __ yes __ no
3. Posters for FMCs __ yes __ no
4. Brochures for patients __ yes __ no
5. Online training combining different FMCs __ yes __ no
6. FMC Pledge __ yes __ no
7. Facebook promotion of staff activities __ yes __ no
8. Other: ___________________________ __ yes __ no

1.5 Of those activities you think should be scaled to other FMCs, identify up to 3 that should be given top priority. *(Interviewer, circle letters that correspond to the activities listed in I.4)*

a. b. c. d. e. f. g. h.

I.6 What recommendations should we give to the Ministry of Health to support breastfeeding- friendly FMCs? *(Interviewer: in conversation ask what things the FMC Director can do to remove barriers and what only MOH can do)*

___________________________________________________________________

## **KNOWLEDGE (K)**

K.1 When ideally should mothers initiate breastfeeding?

__ correct __ incorrect __ didn’t answer

K.2 How long should women breastfeed for:

a. Exclusively? __ correct __ incorrect __ didn’t answer

b. Continued? __ correct __ incorrect __ didn’t answer

K.3 What effects can giving formula or water have on the success of breastfeeding?

__ correct __ incorrect/inadequate __ didn’t answer

K.4 How often should a mother breastfeed her baby in the first two months?

__ correct __ incorrect __ didn’t answer

K.5 What are the signs that her baby is getting enough milk?

__ correct __ incorrect/inadequate __ didn’t answer

K.6 How can a mother increase her milk supply?

K.7 Do you ever show mothers how to position and attach their infants for breastfeeding?

__ yes __ no *[If no, go to K.8]*

K.8 Could you please show me, and describe, how you would teach a mother, by teaching me in your usual way, using a doll, and describing key points?

a. Positioning: __ correct __ incorrect __ didn’t answer

b. Attachment: __correct __ incorrect __ didn’t answer

K.9 What is the most common cause of painful nipples?

__ correct __ incorrect __ didn’t answer

K.10 What is the most common cause of insufficient milk?

__ correct __ incorrect __ didn’t answer

K.11 What are some reasons mothers should stop breastfeeding her baby?

__ correct __ incorrect __ didn’t answer

## **ATTITUDE (A)**

A.1 I am now going to read a list of statements and ask you to tell me how much you agree or disagree with each statement on a scale of 1 to 5 where *1 = strongly disagree, 2 = disagree, 3 = neutral], 4 = agree, 5 = strongly agree. (Interviewer, circle 6 for no answer)*

1. The benefits of breast milk last only as long as the baby is breast fed* 1 2 3 4 5 6
2. Formula feeding is more convenient than breastfeeding 1 2 3 4 5 6
3. Breastfeeding increases mother infant bonding 1 2 3 4 5 6
4. Formula fed babies are more likely to be overfed than breastfed babies 1 2 3 4 5 6
5. Formula feeding is the better choice if the mother plans to go back to work*1 2 3 4 5 6
6. Mothers who formula feed miss one of the great joys of motherhood 1 2 3 4 5 6
7. Women should not breastfeed in public places such as restaurants 1 2 3 4 5 6
8. Breastfed babies are healthier than formula fed babies 1 2 3 4 5 6
9. Breastfed babies are more likely to be overfed than formula fed babies 1 2 3 4 5 6
10. Fathers feel left out if a mother breast feeds* 1 2 3 4 5 6
11. Breast milk is the ideal food for babies 1 2 3 4 5 6
12. Formula is as healthy for an infant as breast milk* 1 2 3 4 5 6
13. Breastfeeding is more convenient than formula 1 2 3 4 5 6

*(key points: items marked with asterisks are reverse-scored.]*

A.2 I am going to show you a card that lists different types of people. In your opinion, who do you think a mother would first turn to for questions or problems about feeding her baby? Who would she turn to next? *(Interviewer, place a rank of 1 and 2 next to the indicated choice)*

___Spouse ___Mother/Mother-in-law ___Friend

___Religious leaders ___Doctor ___Nurses / Midwives

A.3 I am now going to read a list of statements and ask you to tell me how much you agree or disagree with each statement on a scale of 1 to 5 where *1 = strongly disagree, 2 = disagree, 3 = neutral], 4 = agree, 5 = strongly agree. (Interviewer, circle 6 for no answer)*

1. I feel comfortable giving advice to women about breastfeeding 1 2 3 4 5 6
2. Mothers (or their family) do not listen to, or value, my advice* 1 2 3 4 5 6
3. Spending time helping mothers with breastfeeding is very important

compared with my other duties 1 2 3 4 5 6

1. I do not think that my work at the FMC is valuable these days* 1 2 3 4 5 6
2. I get to see the impact my work has on mothers and babies 1 2 3 4 5 6
3. I don’t have the right knowledge or skills to advise about breastfeeding*1 2 3 4 5 6
4. I am given enough support to do my job well 1 2 3 4 5 6
5. My work can make a difference to the health of mothers and babies 1 2 3 4 5 6
6. I don’t have sufficient resources (time/tools) to help mothers breastfeed*1 2 3 4 5 6
7. My goals and the goals of this FMC’s management are well aligned 1 2 3 4 5 6
8. Supporting mothers to breastfeed is not a priority at this FMC* 1 2 3 4 5 6
9. My own personal experience with breastfeeding is more helpful than

formal training or clinical guidelines when I am counseling mothers* 1 2 3 4 5 6

1. I am confident I can help to solve common breastfeeding problems 1 2 3 4 5 6 *(key points: items marked with asterisks are reverse-scored.]*

***END OF INTERVIEW***

------------------------------------------------------------------------------------------------------------------------------------

## **OBSERVATIONS (O)**

O.1 Other comments/observations

*(Interviewer: 1) specifically note if the staff gives an opinion on the impact of COVID-19 on counselling / support offered at the FMC, and/or reports by women of their experience at the hospital during the birth period. 2) Try to record any interesting ‘quotes’ from staff about the intervention or about breastfeeding friendly practices at the FMC.)*

_________________________________________________________________________

_________________________________________________________________________
