# Supplementary material for: Can behavioral science advance breastfeeding-friendly primary care? Key findings from an evaluation in Kosovo
Source: PLOS Glob Public Health. 2025 Oct 31;5(10):e0005276. doi: 10.1371/journal.pgph.0005276 (PMC12578251; doi:10.1371/journal.pgph.0005276)
Supplement: S6 File — (DOCX) [file pgph.0005276.s006.docx]

**S6 File Secondary outcome indicators – detailed tables**

**Mothers’ experience of care**

**Table 1: Mother's rating of her experience at the facility and difference in feelings about breastfeeding after the consultation; a comparison of results before and after intervention period**

|  |  | |  | | **Feelings changed about:** | | | | | |
| --- | --- | --- | --- | --- | --- | --- | --- | --- | --- | --- |
|  | **Rating of overall experience at the facility** | | **‘I believe I can successfully breastfeed my baby’** | | **Value of breastfeeding for my baby's health** | | **Length of time I will breastfeed my baby** | | **My ability to overcome breastfeeding challenges** | |
|  | Before % (N) | After % (N) | Before % (N) | After % (N) | Before % (N) | After % (N) | Before % (N) | After % (N) | Before % (N) | After % (N) |
| Median rating | 3  average | 4  good | 3  moderate | 4  a lot | 2  little | 3  moderate | 2  little | 3  moderate | 2  little | 2  little |
| P value* | <0.001 | | 0.006 | | <0.001 | | <0.001 | | 0.011 | |

**Providers’ knowledge and attitudes**

**Table 2: Provider knowledge before versus after intervention**

|  | **Correct answers as a % of total** | | | |  |
| --- | --- | --- | --- | --- | --- |
|  | **Before** | **n=** | **After** | **n=** | **% pt change** |
| Breastfeeding initiation | 100% | 36 | 99% | 80 | -1.3 |
| Breastfeeding duration: exclusive | 94% | 36 | 100% | 81 | 5.6 |
| Breastfeeding duration: continued | 91% | 35 | 100% | 81 | 8.6 |
| Formula effects | 66% | 32 | 94% | 81 | 28.2 |
| Breastfeeding frequency in first 2 months | 86% | 36 | 96% | 81 | 10.2 |
| Signs baby getting enough milk | 64% | 36 | 100% | 79 | 36.1 |
| How to increase milk supply | 57% | 30 | 95% | 77 | 38.1 |
| Showing mothers positioning/attaching | 94% | 36 | 98% | 80 | 3.1 |
| Demonstration: positioning | 76% | 34 | 95% | 81 | 18.6 |
| Demonstration: attachment | 71% | 34 | 95% | 78 | 24.3 |
| Common cause of painful nipples | 83% | 30 | 94% | 78 | 10.3 |
| Common cause of insufficient milk | 69% | 29 | 92% | 78 | 23.3 |
| Reasons to stop breastfeeding | 75% | 24 | 92% | 78 | 17.3 |
| ***Mean*** | ***80%*** | ***33*** | ***96%*** | ***79*** | ***16.5*** |

**Table 3: Provider attitudes towards breastfeeding: percentage who answered positively^ before intervention versus after intervention (based on an adapted Iowa Infant Feeding Scale(65))**

|  | **Before** | **n=** | **After** | **n=** | **% pt change** |
| --- | --- | --- | --- | --- | --- |
| The benefits of breast milk last only as long as the baby is breast fed* | 59% | 34 | 70% | 81 | 11.5 |
| Formula feeding is more convenient than breastfeeding | 86% | 36 | 95% | 80 | 9.0 |
| Breastfeeding increases mother infant bonding | 92% | 36 | 94% | 78 | 2.2 |
| Formula fed babies are more likely to be overfed than breastfed babies | 33% | 36 | 74% | 78 | 40.7 |
| Formula feeding is the better choice if mother plans to work* | 69% | 36 | 91% | 81 | 21.9 |
| Mothers who formula feed miss one of the great joys of motherhood | 61% | 36 | 85% | 77 | 24.1 |
| Women should not breastfeed in public places such as restaurants* | 71% | 34 | 93% | 81 | 22.0 |
| Breastfed babies are healthier than formula fed babies | 79% | 34 | 91% | 79 | 11.9 |
| Breastfed babies are more likely to be overfed than formula fed babies | 31% | 35 | 43% | 79 | 11.8 |
| Fathers feel left out if a mother breast feeds* | 69% | 35 | 80% | 81 | 11.7 |
| Breast milk is the ideal food for babies | 92% | 36 | 91% | 78 | -0.3 |
| Formula is as healthy for an infant as breast milk* | 86% | 36 | 90% | 81 | 4.0 |
| Breastfeeding is more convenient than formula | 94% | 35 | 91% | 79 | -2.9 |
| ***Mean*** | ***71%*** | ***35*** | ***84%*** | ***79*** | ***12.8*** |
| *reverse-scored ^*strongly agree/agree or strongly disagree/disagree for reverse score questions* | | | | | |

**Table 4: Opinion of influencers: who mother would turn to for questions or problems about feeding her baby (before and after intervention results for providers compared against mothers’ response to the same question at baseline)**

|  | **Before: Staff** | | | **Before: Mothers** | | | **After: Staff** | | |
| --- | --- | --- | --- | --- | --- | --- | --- | --- | --- |
|  | **1st choice** | **2nd choice** | ***Total*** | **1st choice** | **2nd choice** | ***Total*** | **1st choice** | **2nd choice** | ***Total*** |
| Infant's grandmother | 0% | 6% | *6%* | 20% | 31% | *51%* | 35% | 4% | *38%* |
| Doctor | 71% | 21% | *92%* | 62% | 25% | *87%* | 32% | 25% | *57%* |
| Nurse | 26% | 65% | *90%* | 2% | 16% | *18%* | 19% | 57% | *75%* |
| Infant's father | 3% | 6% | *9%* | 13% | 24% | *37%* | 7% | 6% | *14%* |
| Other: internet, friend, religious leader | 0% | 3% | *3%* | 2% | 4% | *6%* | 7% | 9% | *16%* |
| Yellow highlight denotes the two most frequently chosen responses for 1st choice.  Green highlight denotes the two most frequently chosen responses for 2nd choice. | | | | | | | | | |

**Table 5: Provider self-efficacy scores**

|  | **I am comfortable giving BF advice** | **Mothers don't listen/value my advice** | **Supporting BF is important compared to my other duties** | **I don't believe my work is valuable** | **I see the impact of my work on infants/ mothers** | **I don’t have the right knowledge** | **I am given enough support to do my job well** | **My work can make a difference to infants/ mothers** | **I have insufficient resources (time/tools) for BF support** | **My goals are aligned with this facility’s goals** | **BF is not a priority at this facility** | **My personal experience is more valuable than training** | **I am confident I can solve BF problems** |
| --- | --- | --- | --- | --- | --- | --- | --- | --- | --- | --- | --- | --- | --- |
| **After intervention result as % of total responses (percentage point difference to before intervention)** | | | | | | | | | | | | | |
| Strongly disagree | 4% (+1) | **41% (+22)** | 2% (-1) | **14% (-5)** | 6% (+6) | **53% (-14)** | 6% (+3) | 2% (-1) | 10% (-18) | 6% (+6) | **25% (-19)** | **15% (+4)** | 1% (+1) |
| Disagree | 0% (-3) | **20% (-8)** | 10% (+7) | **19% (0)** | 0% (-6) | **19% (+5)** | 15% (+9) | 0% (0) | 9% (-8) | 5% (-3) | **36% (+22)** | **36% (+22)** | 1% (-2) |
| Neutral | 0% (0) | 9% (-13) | 26% (+9) | 32% (+4) | 11% (+8) | 4% (-2) | 41% (+19) | 2% (-4) | 33% (+14) | 32% (+7) | 14% (+6) | 14% (-3) | 2% (-4) |
| Agree | **19% (0)** | 15% (-2) | **31% (0)** | 28% (+14) | **27% (-4)** | 5% (+5) | **20% (+6)** | **36% (+19)** | **35% (+21)** | **40% (+9)** | 12% (+6) | 11% (-8) | **30% (+11)** |
| Strongly agree | **75% (+3)** | 15% (+7) | **27% (-15)** | 6% (-8) | **54% (+1)** | 19% (+13) | **19% (-31)** | **57% (-12)** | **11% (-3)** | **17% (-14)** | 11% (-11) | 25% (-8) | **64% (-3)** |
| No answer | 1% (-2) | 1% (-5) | 4% (-2) | 0% (-6) | 1% (-7) | 0% (-8) | 0% (-6) | 1% (-5) | 2% (-6) | 0% (-6) | 1% (-5) | 0% (-6) | 1% (-5) |
| **Cumulative percentage point difference in ideal response and unideal response after versus before the intervention** | | | | | | | | | | | | | |
| Ideal response | 3 | 14 | -15 | -5 | -3 | -10 | -25 | 7 | 18 | -5 | 3 | 26 | 8 |
| Unideal response | -2 | 5 | 6 | 6 | 0 | 18 | 12 | -1 | -26 | 3 | 5 | -16 | -1 |
| **Bold** denotes what would be the 'ideal' responses. BF= breastfeeding | | | | | | | | | | | | | |
